# Molecular characterization and prevalence of plasmids co-harbouring mcr and ESBL genes

**DOI:** 10.1099/mgen.0.001458

**Published:** 2025-07-29

**Authors:** K.M.G. Houkes, V. Weterings, J.J. Verweij, J.L. Murk, W. van den Bijllaardt, J.J.J.M. Stohr

**Affiliations:** 1 Microvida, Laboratory of Medical Microbiology, Amphia Hospital, Breda, Netherlands; 2 Microvida, Laboratory of Medical Microbiology and Immunology, Elisabeth-TweeSteden Hospital, Tilburg, Netherlands; 3 Department of Infection Prevention and Control, Amphia Hospital, Breda, Netherlands

**Keywords:** Enterobacterales, extended-spectrum beta-lactamase genes, mobile colistin resistance genes, phylogenetic analyses, plasmids

## Abstract

Multidrug-resistant Enterobacterales isolates carrying extended-spectrum beta-lactamases (ESBLs) and mobile colistin resistance (*mcr*) genes pose a significant healthcare threat as they can lead to difficult-to-treat infections. This study investigates the prevalence of isolates co-harbouring ESBL and *mcr* genes and characterizes the plasmids co-harbouring those genes. ESBL-producing Enterobacterales (ESBL-E) isolates identified during point prevalence surveys (PPS) in a Dutch hospital were screened for *mcr* genes. Plasmids co-harbouring *mcr* and ESBL genes were identified using long- and short-read sequencing data, while detecting resistance and replicon genes using AMRFinderPlus and PlasmidFinder. The plasmid database PLSDB was searched for plasmids containing the same *mcr* and ESBL gene(s), and SNP and DCJ-Indel distance analyses were conducted to examine plasmid diversity. The most recent common ancestor (MRCA) was inferred through timed phylogeny analyses in BEAST, and putative composite transposons containing the *mcr* or ESBL genes were identified. Among 188 screened ESBL-E, 11 harboured *mcr* genes: 9 with *mcr-9* and 2 with *mcr-9* and *mcr-4.3*. All plasmids containing *mcr* and ESBL genes were IncHI plasmids harbouring *mcr-9*, *bla*_CTX-M-9_ and/or *bla*_SHV-12_. The plasmid database search resulted in 128 similar plasmids. SNP analysis showed ≤10 SNPs among the PPS study plasmids and up to 924 SNPs among all plasmids. Structural relatedness and phylogenetic analyses revealed clustering of the PPS study plasmids but no additional taxonomical or geographical clustering. The MRCA of the PPS study plasmids likely emerged between 1986 and 2008. Finally, composite transposon analysis indicated that matching complete insertion sequences rarely flanked *mcr-9* genes, whereas *bla*_CTX-M-9_ and *bla*_SHV-12_ frequently were. The prevalence of *mcr* genes among ESBL-E in this study is 5.9%, with *mcr-9* being the most prevalent. Limited plasmid diversity suggests a single introduction event followed by regional and global dissemination across related bacterial species. Persistent ESBL gene mobility suggests a more recent introduction of these genes compared to the *mcr-9* genes.

**Impact Statement**

Colistin is a last-resort antibiotic used to treat serious infections caused by Gram-negative bacteria that are resistant to first-choice antibiotics. An example of such resistant bacteria is extended-spectrum beta-lactamase-producing Enterobacterales (ESBL-E). However, data on the prevalence and characteristics of mobile colistin resistance (*mcr*) genes among ESBL-E are scarce. We provide reliable *mcr* prevalence data among ESBL-E found in patients of a large Dutch hospital over the course of one decade. We characterized all plasmids containing *mcr* and ESBL genes and showed that similar plasmids have been found all over the world. We delve into the plasmid sequences to elucidate plasmid origin and dissemination. Understanding the history of these plasmids that contain multiple antibiotic resistance genes is crucial to curtail their spread and prevent the upsurge of such plasmids in pathogenic Enterobacterales isolates.

## Data Summary

Generated Nanopore read files were submitted to the European Nucleotide Archive of the European Bioinformatics Institute under the study accession number PRJEB85738 with the accession numbers given in Table S1, accompanied by the accession numbers of the previously published matching Illumina reads.

## Introduction

Colistin is considered a last-resort antibiotic for treating Gram-negative bacterial infections and is classified by the World Health Organization as a Critically Important Antimicrobial for Human Medicine [1]. The emergence of mobile colistin resistance (*mcr*) genes, first identified in China in 2015, has raised significant global concerns [2]. MCR proteins are phosphoethanolamine transferase enzymes that modify the bacterial cell wall and are the primary mechanism for colistin resistance [3]. The *mcr-1* gene was the first plasmid-mediated colistin resistance gene reported and was soon followed by the discovery of nine homologs: *mcr-2* to *mcr-10* [3]. The *mcr* genes have been extensively studied and found on various conjugative plasmids. They are widespread throughout the world across different bacterial species, and it is believed that each *mcr* gene has a distinct origin. For example, *mcr-1* and *mcr-2* are thought to have originated from intrinsic *mcr*-like genes of *Moraxella* species, while *mcr-9* and *mcr-10* likely found their origin on the chromosome of *Buttiauxella* species [3].

The emergence and global dissemination of *mcr* genes has drawn significant attention from the ‘One Health’ perspective, as the use of colistin in livestock is thought to play a key role in their dissemination, facilitating spillover through the food chain to clinically relevant Enterobacterales infecting humans [3]. A particularly concerning development is the co-occurrence of *mcr* genes and extended-spectrum beta-lactamase (ESBL) genes, particularly CTX-M-type ESBLs, on plasmids [4,6]. This confers drug resistance to polymyxins and third-generation cephalosporins, critical treatments for Gram-negative infections. Knowledge of the prevalence, diversity and dissemination routes of the plasmids with ESBL and *mcr* genes is urgently needed to curb further spread.

This study describes the prevalence of *mcr* genes among ESBL-producing Enterobacterales (ESBL-E) found during routine screening for ESBL-E rectal carriage in a Dutch hospital and the characteristics of plasmids co-harbouring *mcr* and ESBL genes. We hypothesized that the regional distribution of resistance genes resulted from clonal expansion of a single plasmid that obtained *mcr* and ESBL genes. To investigate this, we compared the plasmids from our study with previously published sequences of plasmids carrying the same *mcr* and ESBL genes. Plasmid diversity was assessed through replicon typing and SNP analyses. Additionally, inferred timed phylogeny was applied to estimate the timing of the origin of these plasmids. Finally, our study aimed to assess the order of events in which the *mcr* and ESBL genes became part of the plasmids of our study by identifying putative composite transposons.

## Methods

### Sample selection and study population

Annual point prevalence surveys (PPS) to screen for ESBL rectal carriage in hospitalized patients were conducted from 2013 to 2022 as part of the infection control policy at a Dutch teaching hospital [7]. Whole genome short-read sequencing data from all 188 ESBL-E isolates identified during this PPS study were previously described by Houkes *et al*. [7]. The sequence data of the 188 isolates were screened for *mcr* genes using AMRFinderPlus v3.12.8 with the plus option [8]. Isolates containing both *mcr* and ESBL genes were included in the analysis. Clonal relatedness among the Enterobacterales of the same species detected within the same year was determined based on species-specific whole genome multi-locus sequence typing schemes using Ridom SeqSphere+ version 8.0 (Ridom GmbH, Munich, Germany). Clonal relatedness was defined as fewer than ten allelic differences. For each group of clonally related isolates, one random isolate was long-read sequenced.

### Culturing and susceptibility testing

Isolates selected for long-read sequencing were cultured from the −70 °C storage at 35 °C for 18–24 h on ESBL screening agars, consisting of a split MacConkey agar with each side containing either ceftazidime (1.0 mg l^−1^) or cefotaxime (1.0 mg l^−1^) (AlphaOmega, ‘s-Gravenhage, Netherlands) to prevent plasmid loss. Colistin susceptibility testing was performed using broth microdilution Micronaut MIC-strip Colistin as per the manufacturer’s protocol (Merlin Diagnostika GmbH, Bornheim, Germany).

### DNA isolation and long-read sequencing

DNA isolation was performed using the Gentra Puregene Kit according to the manufacturer’s instructions (Qiagen, Hilden, Germany). Long-read sequencing data were obtained using a MinION sequencer with either an R9.4.1 flow cell with the Rapid Barcoding Kit 12 or an R10.4.1 flow cell with the Rapid Barcoding Kit 96 V14, including the optional AMPure XP beads purification step according to the standard protocol provided by the manufacturer (Oxford Nanopore Technologies, Oxford, United Kingdom).

### Hybrid assembly and *mcr*-containing plasmid identification

A hybrid assembly was performed using Unicycler v0.4.8 with default parameters [9]. If the Nanopore read file sizes were >3 gigabytes, Filtlong v0.2.1 (R. Wick, https://github.com/rrwick/Filtlong) was used to filter the long reads by quality using the default settings and the Illumina reads as reference. Genetic components were extracted from the created Graphical Fragment Assembly files using a Python script (supplementary methods). The presence of *mcr* genes and other resistance genes in all components was assessed using AMRFinderPlus v3.12.8 with the plus option [8]. For further analysis, only complete plasmids containing both *mcr* and ESBL genes were included and referred to as PPS study plasmids.

### Public database search for *mcr* and ESBL gene-containing plasmids

The *mcr* genes present on the *mcr* and ESBL gene-containing PPS plasmids were blastn searched (identity >90%, query coverage >90%) in the plasmid database PLSDB v. 2023_11_03_V2 [10,11] (via https://ccb-microbe.cs.uni-saarland.de/plsdb/). The available metadata for all hits was downloaded, including the nuccore accession number, creation date, geographical location of isolation, isolation source, host, plasmid length, G+C content and bacterial species. FASTA files for these hits were extracted from the FASTA plasmid archive using a Python script (supplementary methods).

### Plasmid characteristics analysis

Plasmids containing an *mcr* and ESBL gene detected in the PPS study and from the public database were annotated using the Bakta v1.5.1 database v4.0 [12], which includes the ISFinder [13] and AMRFinder [14] databases. Plasmid replicon genes were identified using Abricate 1.0.1 (T. Seemann, https://github.com/tseemann/abricate) with the PlasmidFinder database updated in July 2024 [15]. Mobility/relaxase type was determined using Mob-suite v3.1.0 [16].

### Plasmid selection

All plasmids with a complete assembly and containing both *mcr* and ESBL genes detected in the PPS study were included. Extracted database plasmids were only included when they belonged to the same replicon type as the PPS study plasmids and when they contained the same *mcr* and ESBL genes as the PPS study plasmids. The presence of *mcr* and ESBL genes on the extracted database plasmid sequences was assessed using the AMRFinderPlus v3.12.8 with the plus option [8]. If multiple ESBL genes were present, at least one of these ESBL genes must be the same as found on the PPS study plasmids. Thus, only the database plasmids resembling the PPS study plasmids were selected for further analyses.

### Plasmid comparison

The plasmids containing both *mcr* and ESBL genes from the PPS study, along with plasmids from the database harbouring the same *mcr* and ESBL genes, were grouped based on their replicon genes and analysed by group.

SNP differences were assessed using Snippy 4.6.0, with the most recently isolated plasmid from the PPS study as the reference genome [17]. The full core SNP alignment was used to generate the phylogenetic tree with RaxML-NG v1.2.2 [18]. The substitution model was selected based on the best Bayesian information criterion among all possible substitution models evaluated in the model fitting test ModelTest-NG v0.1.7 [19]. The phylogenetic trees were calculated using 10 runs and bootstrapping 1,000 times. The best tree was visualized using iTOL v7.0 [20]. For assessing the structural relatedness between the plasmids, pling v2.0.0 with default parameters was used [21]. The subcommunity containing the PPS study plasmids was visualized using Cytoscape v3.10.3 [22].

### Phylogenetic dating

Bayesian-based molecular dating approaches were used to determine the timing of the most recent common ancestor (MRCA). First, the best tree from the phylogenetic analysis was used to identify regions of high recombination using ClonalFrameML [23]. BEAST2 v2.7.7 [24] was used to infer a timed phylogeny from the plasmids with the same replicon type after the removal of regions with evidence of high recombination. Aligned sequences were annotated with their respective sampling dates as recorded in GenBank or corresponding publications. Plasmids with unknown sampling dates were excluded from these analyses. Three population size models were used in BEAST2 analyses with default settings: coalescent constant population, coalescent exponential population and coalescent Bayesian skyline with loose default priors. All analyses were performed using the HKY substitution model prior with either a strict clock or relaxed clock prior. For the analyses with a strict clock prior on the molecular clock, a range from 1E-8 to 0.01 with a log-normal distribution was used, and for the analyses with a relaxed clock, the optimized relaxed clock default settings were selected. The Markov Chain Monte Carlo (MCMC) analyses ran for 2.5×10^7^ iterations for analyses with a strict clock and for 4.0×10^8^ iterations for analyses with a relaxed clock to ensure convergence. All effective sample sizes (ESS) of the posterior distributions were evaluated after discarding the first 10% of iterations as burn-in, using Tracer v1.7.2. Runs were deemed successful if they converged with ESS of the posteriors >200. The time-annotated maximum clade credibility (MCC) tree was generated using the sampled posterior trees of the different MCMC analyses using TreeAnnotator v2.7.7, keeping target heights as node heights and visualized in FigTree v1.4.4 (http://tree.bio.ed.ac.uk/software/figtree/).

### Transposon identification

Putative transposons containing the *mcr* and ESBL genes of interest were identified by searching for matching insertion sequences (ISs) and transposases in the annotated plasmids. These analyses can provide clues about gene mobility and the order in which the genes were incorporated into the plasmid. The presence of complete matching ISs points towards a recent introduction of the gene into the plasmid [25]. A putative composite transposon was defined when matching IS/transposases were located upstream and downstream of the *mcr* and/or ESBL gene of interest within <50 Kb. The length of the composite transposon was calculated as the distance from the start of the upstream IS/transposase to the end of the downstream IS/transposase. In the case of multiple matching transposases, the shortest distance was reported. If no matching IS/transposases were found within the specified range, the nearest IS/transposases up- and downstream of the gene were listed.

## Results

### Plasmids with *mcr* and ESBL genes

Out of 188 isolates containing an ESBL gene, 11 isolates (5.9%) also harboured an *mcr* gene. Nine of these isolates belonged to the *Enterobacter cloacae* complex. Nine isolates carried an *mcr-9* gene and two isolates contained *mcr-4.3* and *mcr-9* genes. Three pairs of clonally related isolates were identified (6, 7 and 1 allelic difference between them), resulting in eight unique isolates that were selected for long-read sequencing and colistin minimal inhibitory concentration (MIC) testing (Table 1).

**Table 1.** PPS study isolates containing both *mcr* and ESBL genes

Isolate	Species	Year of collection	ESBL gene	Other beta-lactamase	*mcr* gene	Colistin MIC (mg l^−1^)	Hybrid assembly
**A1401**	*E. cloacae* complex	2014	*bla*_SHV-12_, *bla*_CTX-M-9_	*bla*_ACT-67_	*mcr-9.2*	0.25	Complete
**A1402**	*Citrobacter freundii*	2014	*bla*_CTX-M-9_	*bla*_TEM-1_, *bla*_CMY-150_	*mcr-9.2*	0.5	Complete
**A1501**	*E. cloacae* complex	2015	*bla*_SHV-12_, *bla*_CTX-M-9_	*bla*_ACT-67_	*mcr-9.2*	0.125	Complete
**A1701**	*E. cloacae* complex	2017	*bla*_SHV-12_, *bla*_CTX-M-9_	*bla*_ACT-67_	*mcr-9.2*	0.25	Complete
**A1702**	*E. coli*	2017	*bla*_CTX-M-9_	*bla*_EC15_	*mcr-9*	0.25	Complete
**A1901**	*E. cloacae* complex	2019	*bla*_SHV-12_, *bla*_CTX-M-9_	*bla*_ACT-67_	*mcr-9.2*	0.25	Complete
**A2101**	*E. cloacae* complex	2021	*bla*_CTX-M-15_	*bla*_OXA-1_, *bla*_TEM-1_, *bla*_ACT-64_	*mcr-9*, *mcr-4.3*	16	Complete
**A2201**	*E. cloacae* complex	2022	*bla*_SHV-12_, *bla*_CTX-M-9_	*bla*_ACT-24_	*mcr-9.2*	0.5	Incomplete

The colistin MIC for isolates containing only an *mcr-9* gene ranged from 0.125 to 0.5 mg l^−1^. The colistin MIC was 16 mg l^−1^ for *E. cloacae* isolate A2101 containing *mcr-4.3* and *mcr-9* genes (Table 1). The hybrid assemblies of the eight selected isolates revealed one *mcr* gene-containing plasmid per isolate (Table 2). In *E. cloacae* isolate A2101, the *mcr-9* gene was located on the chromosome. However, this isolate harboured a ColE10 plasmid with an *mcr-4.3* gene without additional resistance genes. All seven plasmids containing *mcr-9* also carried ESBL genes (*bla*_CTX-M-9_ and/or *bla*_SHV-12_). These *mcr-9-* and ESBL-gene-containing plasmids belonged to the IncHI2 group with relaxase type MOBH and were included for further analysis (Table 2).

**Table 2.** Identified *mcr* gene-containing plasmids and their characteristics

Plasmid	Isolate	Length (bp)	Genes (No.)	Replicon	Mobility type	ESBL gene	*mcr* gene
**pMCRESBL1**	A1401	268.712	294	IncHI2A/IncHI2	MOBH	*bla*_SHV-12_, *bla*_CTX-M-9_	*mcr-9.2*
**pMCRESBL2**	A1402	242.886	263	IncHI2A/IncHI2	MOBH	*bla*_CTX-M-9_	*mcr-9.2*
**pMCRESBL3**	A1501	304.453	335	IncHI2A/IncHI2	MOBH	*bla*_SHV-12_, *bla*_CTX-M-9_	*mcr-9.2*
**pMCRESBL4**	A1701	282.195	315	IncHI2A/IncHI2	MOBH	*bla*_SHV-12_, *bla*_CTX-M-9_	*mcr-9.2*
**pMCRESBL5**	A1702	242.975	261	IncHI2A/IncHI2	MOBH	*bla*_CTX-M-9_	*mcr-9*
**pMCRESBL6**	A1901	274.716	307	IncHI2A/IncHI2	MOBH	*bla*_SHV-12_, *bla*_CTX-M-9_	*mcr-9.2*
**pMCRESBL7**	A2101	12.808	14	ColE10	MOBQ	*None*	*mcr-4.3*
**pMCRESBL8**	A2201	278.511	311	IncHI2A/IncHI2	MOBH	*bla*_SHV-12_, *bla*_CTX-M-9_	*mcr-9.2*

To compare the included PPS study plasmids to already published plasmids carrying the same genes, a database search was conducted using the *mcr-9* reference sequences (*mcr-9.1* and *mcr-9.2*). This resulted in 268 plasmids, of which 142 (53.0%) also harboured an ESBL gene (Table S2, available in the online Supplementary Material). The *bla*_SHV-12_ and *bla*_CTX-M-9_ were most abundant and present in 74.0 (*n*=105) and 38.7% (*n*=55) of the plasmids, respectively, together accounting for 130 plasmids containing *bla*_SHV-12_ and/or *bla*_CTX-M-9_.

**Fig. 1.**
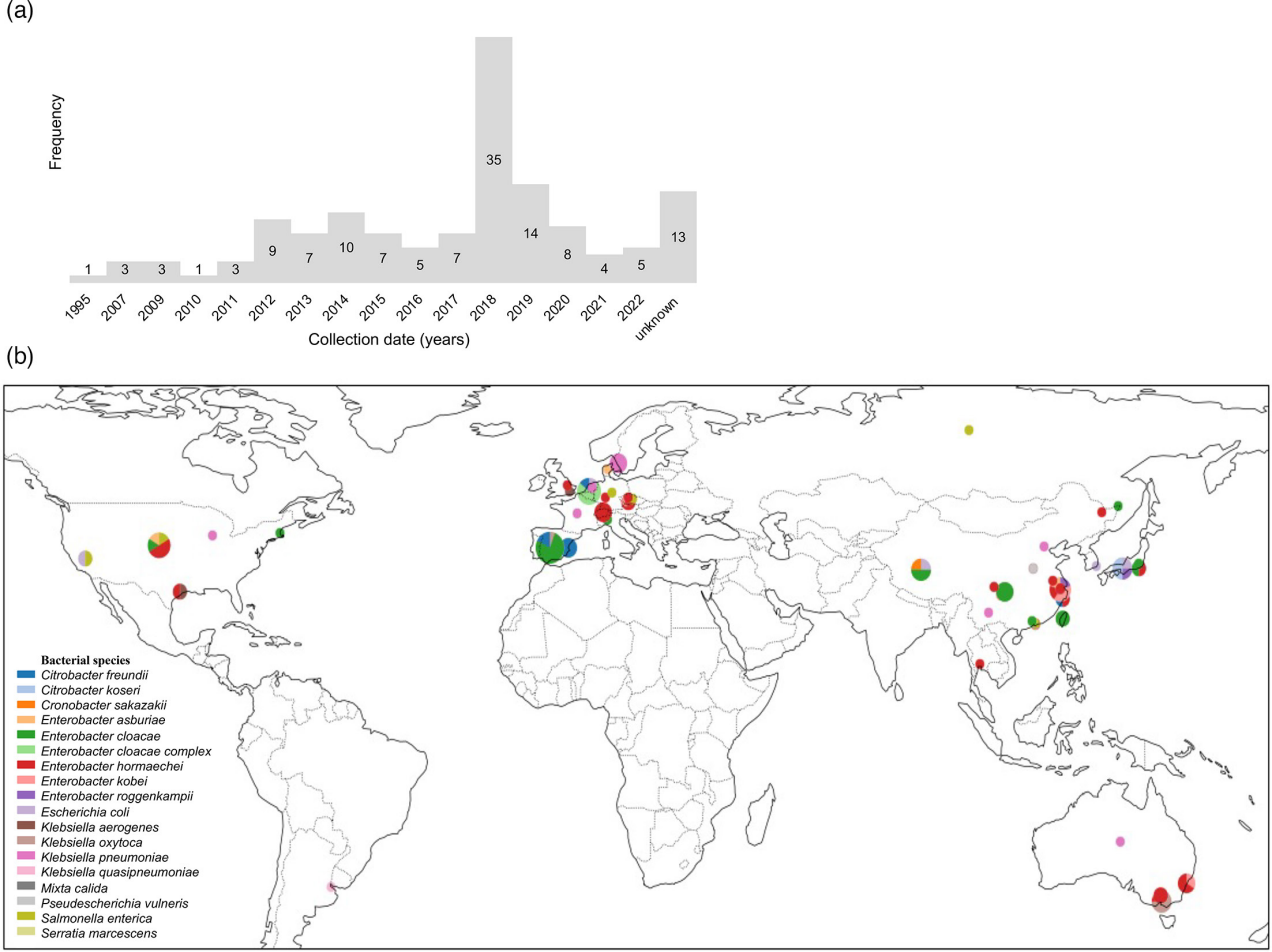
Overview of the seven PPS study plasmids and the 128 database plasmids containing *mcr-9*, along with *bla*_SHV-12_, *bla*_CTX-M-9_ or both. (a) Absolute number of isolates identified each year. (b) Global map showing the collection location of the included isolates. The colours in the pie charts represent the different species, and the size of each pie chart indicates the sample size per location.

For further analysis, 128 plasmids (47.8%) resembling those from the PPS study, defined as plasmids containing *mcr-9* along with *bla*_SHV-12_ and/or *bla*_CTX-M-9_ belonging to the IncHI group*,* were extracted from the database (referred to as database plasmids) (Table S3). Similar to the PPS study plasmids, the majority of the database plasmids were isolated from *Enterobacter* species, 71.4 (*n*=5) and 65.6% (*n*=84), respectively. The PPS study plasmids were all collected between 2014 and 2022, like most of the database plasmids (79.7%, *n*=102) (Fig. 1a). Geographically, the PPS study plasmids were all found in the Netherlands, while the isolation of the database plasmids was globally distributed (Fig. 1b).

### Comparison of plasmids

**Fig. 2.**
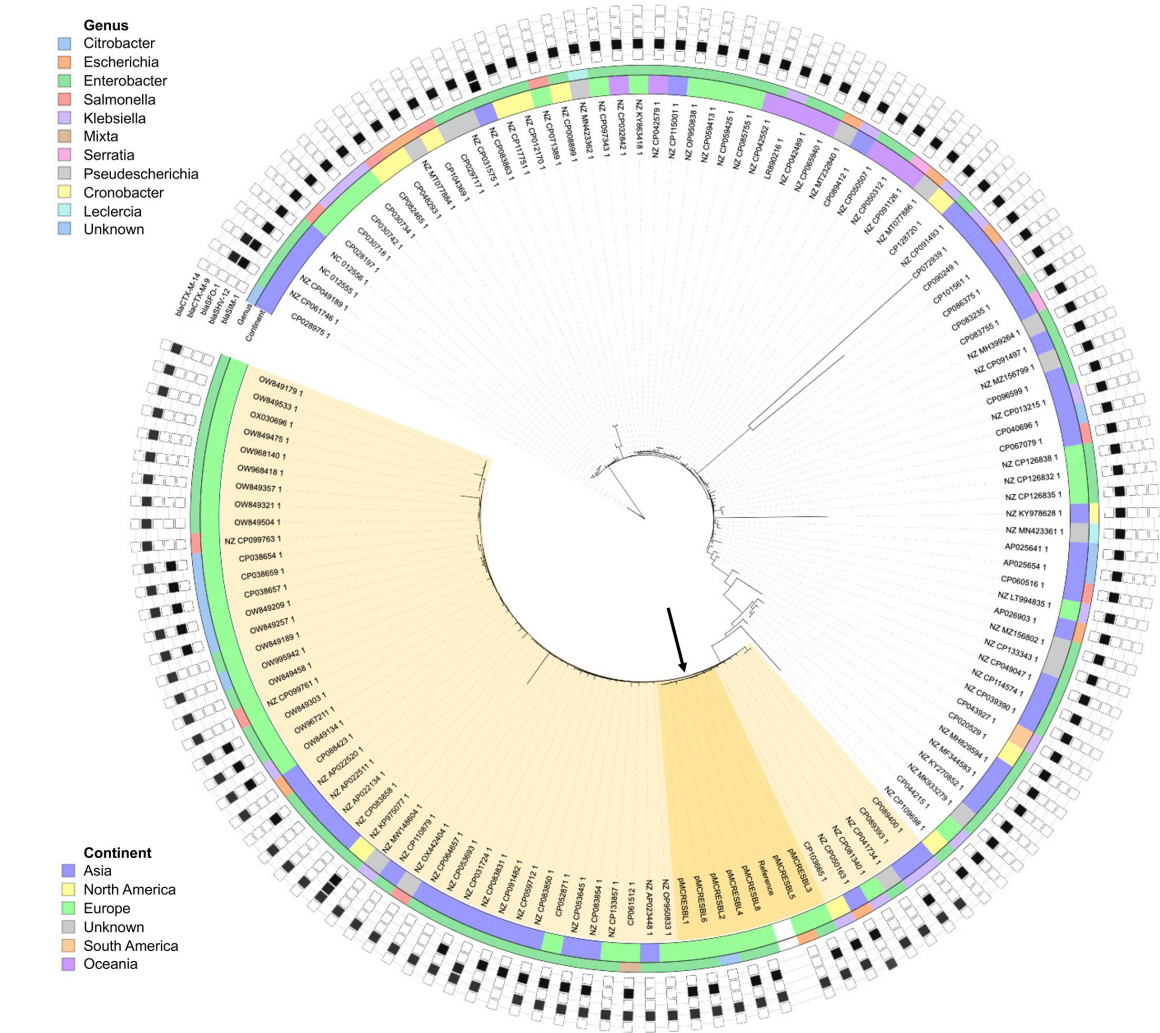
Maximum likelihood tree of all IncHI plasmids encoding *mcr-9* co-localized with *bla*_CTX-M-9_ and/or *bla*_SHV-12_. The colour of the inner circle depicts the continent where the isolate was found, the colour of the middle circle shows the genus of the isolate and the outer circle indicates whether the listed ESBL genes were present on the plasmid (*bla*_CTX-M-14_, *bla*_CTX-M-9_, *bla*_SFO-1_, *bla*_SHV-12_ and *bla*_SIM-1_). The plasmids of the PPS study are highlighted in dark yellow and indicated with an arrow, and the more closely related isolates (<80 SNPs of at least one of the PPS study plasmids) in light yellow.

The median number of SNPs representing the number of mutations between the plasmids of the PPS study was seven (range: 3–10 SNPs). In comparison, the SNP differences between the database plasmids ranged from 1 to 924, with a median of 105 SNPs. Phylogenetic analysis based on maximum likelihood using the substitution model HKY+I+G4 revealed that the plasmids identified in the present study clustered closely together based on SNP differences, as depicted in dark yellow in Fig. 2. The plasmids from the PPS study differed by 7–80 SNPs when compared to a subset of plasmids from North America, Asia and other European countries, highlighted in light yellow in Fig. 2. There was no distinct overall geographical or taxonomical clustering in the maximum likelihood tree. Comparative structural analysis of the PPS study plasmids using Pling showed that these plasmids are separated by 2–8 structural events with a median of 4, all belonging to the same subcommunity together with 17 database plasmids (Fig. S1).

### Phylogenetic dating

**Fig. 3.**
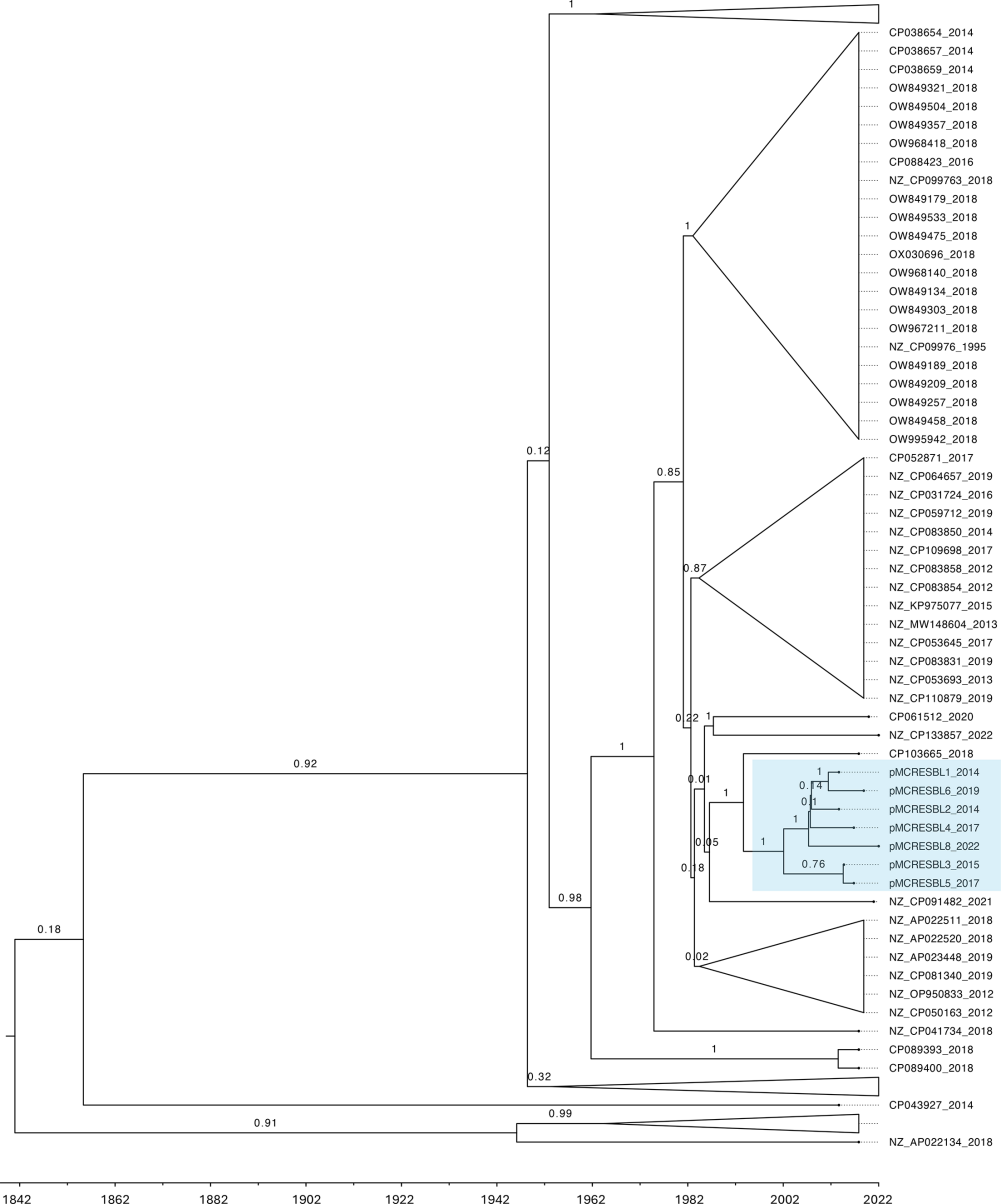
Part of the time-scaled MCC tree is based on inferred time phylogeny according to the coalescent Bayesian skyline model with an optimized relaxed clock of all IncHI plasmids containing *mcr-9*, *bla*_SHV-12_ and/or *bla*_CTX-M 9_. The PPS study plasmids are highlighted in blue, and the branches are annotated with the posterior probability. The time in years at the bottom of the tree represents the year of the origin of the nodes. The full MCC tree can be found in Fig. S8.

The year of origin of the MRCA of the PPS study and database plasmids was predicted using inferred timed phylogeny. Excluded from these analyses were the 13 plasmids without information on the sampling dates and the plasmid regions suspected of recombination (47,338 bp in total), resulting in an alignment of 231,173 bp across 122 IncHI plasmids (Fig. S2). All tested models converged with ESS of the posteriors >200. Based on a Bayesian dating approach with the six combinations of model and clock settings tested, the MRCA of the PPS study plasmids arose between 1986 and 2008 (Table 3). The MRCA of all included IncHI plasmids arose between 1827 and 1931 (Table 3). A mutation rate of around 1×10^−6^ substitutions per site per year was found in the models with a strict clock (Table S4). The time-scaled MCC trees show similar topology across all models but differ in the divergence times of the different branches (Figs 3 and S3–S8).

**Table 3.** The estimated years in which the MRCA likely arose according to the different models for the PPS study plasmids and the PPS study plasmids together with the similar plasmids found in the database

			Estimated date of MCRA (year)	
**Plasmids**	**Model**	**Clock**	**Median**	95% HPD*
PPS study plasmids (pMCRESBL1-6, 8)	Coalescent constant population	Strict	2007.6	2001.9–2011.8
		Relaxed	2005.0	1993.2–2011.6
	Coalescent exponential population	Strict	2004.5	1996.7–2010.2
		Relaxed	1986.4	1907.1–2009.1
	Coalescent Bayesian skyline	Strict	2004.3	1974.0–2012.5
		Relaxed	1993.0	1,188 BCE–2011.5
All IncH plasmids containing *mcr-9*, *bla*_SHV-12_ and/or *bla*_CTX-M-9_	Coalescent constant population	Strict	1908.1	1864.8–1941.8
		Relaxed	1827.0	1635.8–1931.1
	Coalescent exponential population	Strict	1930.8	1896.0–1955.9
		Relaxed	1893.5	1581.6–1970.6
	Coalescent Bayesian skyline	Strict	1902.1	1857.5–1937.8
		Relaxed	1841.4	17,527 BCE–1970.2

*95% HPD=95% highest posterior density.

### Direct genomic background of *mcr-9*, *bla*_SHV-12_ and/or *bla*_CTX-M-9_ genes

In the PPS study plasmids, *bla*_CTX-M-9_ was located on a putative composite transposon in six plasmids (85.7%), all flanked by Tn3 and with a median length of 18,408 bp. The *bla*_SHV-12_ gene was found on a putative composite transposon flanked by IS*6* in all cases (*n*=5), with a median length of 7,037 bp. Notably, in three cases, this transposon was situated within the Tn3-flanked transposon containing *bla*_CTX-M-9_. In contrast, the *mcr-9* gene was only located on a putative composite transposon on one occasion, which was flanked by Tn3 with a length of 22,414 bp. In all seven cases, the *mcr-9* gene was directly flanked by IS*1* upstream and IS*5* downstream. In none of the PPS study plasmids, the *mcr-9* gene and ESBL gene(s) were found within the same composite transposon.

Among the database plasmids, *bla*_CTX-M-9_ was located on a composite transposon in 63.0% (34/54), ranging from 13 to 23 Kb in length and mainly flanked by Tn3 (*n*=31, 91.2%) (Fig. S9). The *bla*_SHV-12_ was located on a putative composite transposon in 80.8% (84/104) plasmids, flanked by IS*6* in all cases, with a total length of <10 Kb. In the database plasmids, the *mcr-9* gene was found on a putative composite transposon in 28.9% (37/128) plasmids (Fig. S9). The majority of these transposons were flanked by IS*5* (*n*=21, 56.8%) or IS*1* (*n*=12, 32.4%).

## Discussion

The prevalence of *mcr* gene-containing ESBL-E from peri-anal swabs from patients admitted at our Dutch hospital was 5.9% (*n*=11). The *mcr* genes found were *mcr-4.3* and *mcr-9*, with the latter being most abundant and present in all 11 isolates. These isolates were collected between 2013 and 2022. An *mcr* prevalence of 4.2–7.4% among healthy humans and clinical isolates was found in a meta-analysis of the global *mcr-*mediated colistin resistance among *Escherichia coli* isolates, including data from the Netherlands [26]. Regarding ESBL-producing isolates, no previous report on the *mcr* prevalence in the Netherlands was published. A recent report from Cambodia reported a prevalence of 8.5% among clinical *E. coli* and *Klebsiella pneumoniae* ESBL-producing isolates [27]. The relative abundance of *mcr-9* genes in our study aligns with the status of *mcr-9* as one of the most prevalent subtypes of the *mcr* family worldwide and its previous association with ESBL-producing isolates and plasmids [28,30].

The clinical significance of *mcr-9* remains debated since isolates carrying this gene typically exhibit low MICs for colistin, often within the susceptible range [3,28]. Although this might make the clinical significance of *mcr-9* less than for other *mcr* homologues, clinically relevant isolates with high colistin MICs due to high expression levels of *mcr-9* have been described [28]. Notably, exposure to subinhibitory concentrations of colistin can upregulate *mcr-9* expression, leading to colistin resistance [3,31]. This could render colistin ineffective against *mcr-9*-containing isolates *in vivo*, while having low MICs for colistin *in vitro*, making them difficult to detect using conventional methods [3,28]. More research on this potential phenomenon is needed.

We identified seven plasmids co-harbouring *mcr* and ESBL genes. These were all IncHI plasmids containing *mcr-9* and *bla*_CTX-M-9_ with an additional *bla*_SHV-12_ gene in five plasmids. That the plasmids all belong to the same replicon type and differ maximally by ten SNPs from each other supports our hypothesis of a single introduction of an *mcr* gene and ESBL gene into the same plasmid with subsequent spread in our region. The plasmids in the database containing *mcr-9* and *bla*_CTX-M-9_ or *bla*_SHV-12_ or both also mostly belonged to the replicon group IncHI (98.5%). Compared to our plasmids, these database plasmids had a median difference of 105 SNPs despite being isolated within a period of 27 years and collected from all over the world. This further supports our hypothesis of a single introduction of these genes onto one plasmid, followed by subsequent global spread. Moreover, the MRCA of the seven PPS study plasmids was estimated to have originated between ~1986 and 2008, which indicates that it took ~15–35 years to arrive at a prevalence of 5.9% in the period from 2013 to 2022. If the dissemination continues at this rate, Gram-negative bacteria harbouring these plasmids could pose a serious threat to human health.

In the search for composite transposons to investigate gene mobility and the order of events in which the genes were incorporated into the plasmids, we found that both investigated ESBL genes, *bla*_CTX-M-9_ and *bla*_SHV-12_, were mostly flanked by intact ISs, enabling transposon-mediated gene mobility. Interestingly, in three cases, the *IS6*-flanked composite transposon containing *bla*_SHV-12_ was situated within the Tn3-flanked composite transposon containing *bla*_CTX-M-9_, enabling joint gene mobility. The possibility of joint transposon-mediated gene mobility of an ESBL gene with an *mcr* gene was not observed. In most cases, transposon-mediated gene mobility of *mcr-9* seemed unlikely, as no matching intact ISs flanking these genes were found. This suggests that *bla*_CTX-M-9_ and *bla*_SHV-12_ were more recently introduced into the plasmid background than *mcr-9*, as the ISs that initially transferred *mcr-9* onto the plasmid likely got lost over time, which has not yet occurred for *bla*_CTX-M-9_ and *bla*_SHV-12_.

Here, we conducted an in-depth characterization of plasmids co-harbouring *mcr* and ESBL genes from a selection of clinical isolates. Unlike many studies on *mcr* prevalence that begin with phenotypic screening using colistin MICs, we employed molecular techniques to directly identify these genes regardless of colistin susceptibility [32]. This approach allowed us to detect all *mcr* gene-containing isolates, including *mcr-9*, *mcr-3* and *mcr-5*, which have been identified in isolates exhibiting low colistin MICs [33]. We provided a comprehensive overview of the prevalence of *mcr* gene-containing isolates and the plasmids harbouring both ESBL and *mcr* genes. However, further research is needed to elucidate the transmission dynamics of these plasmids and isolates. A deeper understanding of these mechanisms is crucial to anticipate and mitigate the potential threats they pose to human health.

## Supplementary material

10.1099/mgen.0.001458 Uncited Supplementary Material 1.

## Abbreviations

ESBL: extended-spectrum beta-lactamase
ESBL-E: ESBL-producing Enterobacterales
ESS: effective sample size
IS: insertion sequence
MCC: maximum clade credibility
MCMC: Markov Chain Monte Carlo
MCR: mobile colistin resistance
MRCA: most recent common ancestor
PPS: point prevalence surveys
