# Supplementary material for: Molecular characterization and prevalence of plasmids co-harbouring mcr and ESBL genes
Source: Microb Genom. 2025 Jul 29;11(7):001458. doi: 10.1099/mgen.0.001458 (PMC12306943; doi:10.1099/mgen.0.001458)
Supplement: Uncited Supplementary Material 1. [file mgen-11-01458-s001.pdf]

Supplementary methods

Python script 1 – gfa file components to separate fasta files

```
from collections import defaultdict
from glob2 import glob
import pandas as pd
import os

# This class represents a directed graph using adjacency list representation from typing import List
class Graph:

    def __init__(self):
        self.graph = defaultdict(list)
    def addEdge(self, u, v):
        self.graph[u].append(v)

    def BFS(self, s):
        list = []
        visited = [False] * (max(self.graph) + 1)
        queue = []
        queue.append(s)
        visited[s] = True
        while queue:
            s = queue.pop(0)
            list.append(int(s))
            for i in self.graph[s]:
                if visited[i] == False:
                    queue.append(i)
                    visited[i] = True
        return list

# Create a graph given in the above diagram

class reader:

    def __init__(self):
        return

    def read_files(self, file):
        nodes = []
        edges = []
        with open(file) as f:
            for line in f:
                if line.startswith("S"):
                    l = line.split("\t")
                    nodes.append(l[1])
                elif line.startswith("L"):
                    l = line.split("\t")
                    edge = []
                    edge.append(l[1])
                    edge.append(l[3])
                    edges.append(edge)
                else:
                    continue
```

```
        lst3 = [value for value in nodes if value not in edges]
        for node in lst3:
            if None:
                break
            else:
                extra = []
                extra.append(node)
                extra.append(node)
                edges.append(extra)
        unique_edges = [list(x) for x in set(tuple(x) for x in edges)]
        return nodes, unique_edges

reading = reader()
list_of_files= glob("Directory_gfa_files/*.gfa")
component_dict = []
for file in list_of_files:
    new_file = file.replace("Ouput_directory/", "")
    nodes, edges = reading.read_files(file)
    components = []

    g = Graph()
    for edge in edges:
        g.addEdge(int(edge[0]), int(edge[1]))
        g.addEdge(int(edge[1]), int(edge[0]))

    for node in nodes:
        lijst = g.BFS(int(node))
        lijst = sorted(lijst)
        components.append(lijst)

    components = [list(x) for x in set(tuple(x) for x in components)]
    dt_name_nodes = {new_file : components}
    component_dict.append(dt_name_nodes)
print(component_dict)

frames = []
for dict in component_dict:
    for key in dict.keys():
        teller = 0
        for contig in dict[key]:
            teller = teller + 1
            df = pd.DataFrame(contig, columns=["Contig"])
            df["Component"] = teller
            df["Assembly"] = key
            frames.append(df)

df_component_list = pd.concat(frames)
print(df_component_list)

# Dataframe to excel
df_component_list.to_excel("Output_directory_component_list.xlsx")

# Save each components as fasta files
def get_fasta(contig, file):
    fasta = ""
    with open(file) as f:
        for line in f:
```

```
            if "S\t" + str(contig) in line:
                l = line.split("\t")
                fasta += str(l[2])
    return fasta

output_dir = "Output_directory"
os.makedirs(output_dir, exist_ok=True)

list_of_files = glob("Directory_gfa_files/*.gfa")
print("List of GFA files:", list_of_files)

for file in list_of_files:
    gfa_file = file.replace("Output_directory/", "")
    key = gfa_file
    print("Processing GFA file:", key)
    for dict_entry in component_dict:
        if key in dict_entry:
            # selectie file naam in dictonary als key en ga alle bijbehorende contig nummers af
            for contig_list in dict_entry[key]:
                contig_list_name = '_'.join(map(str, contig_list))
                output_file_path = f”Output_directory/{key}_{contig_list_name}.fasta"
                with open(output_file_path, "a") as output_file:
                    for contig in contig_list:
                        code = get_fasta(contig, file)
                        header = f"{key} {contig}"
                        contig = f"{header}\n{code}\n"
                        output_file.write(contig)
                print(f"Finished writing to {output_file_path}")

import os
import subprocess

directory_path = " Output_directory"

for file in os.listdir(directory_path):
    if file.endswith(".fasta"):
        new_name = file.replace("_assembly.gfa","")
        old_path = os.path.join(directory_path, file)
        new_path = os.path.join(directory_path, new_name)
        subprocess.run(f"cp {old_path} {new_path}", shell=True)
        os.remove(old_path)
```

Python script 2 – search plasmids form table in plasmid database and save as fasta file

```
import pandas as pd

#Search for all IDs in excel file the matching fasta sequence in plsdb.fna
file = 'Directory_with_all_record_to_search_for_in_the_plasmiddatabase.xlsx'

df = pd.read_excel(file)
PlasmidIDs = df.iloc[:, 1]
ID_list = PlasmidIDs.tolist()
print(ID_list)

for ID in ID_list:
    sequence = ""
    found = False
```

```
db = ' Plasmiddatabase/plsdb.fna'
with open(db) as f:
  for line in f:
    # Check if the line contains the ID
    if line.startswith(">" + ID):
      found = True
      sequence = ""  # Reset the sequence for a new ID
    elif found:
      if line.startswith(">"):
        break  # Stop if the next sequence header is encountered
      sequence += line.strip()
    # Print the sequence in FASTA format if the ID was found
  if found:
    fasta = f'>{ID}\n{sequence}\n'
    output_file_path = f"/Output_directory/{ID}.fasta"
    with open(output_file_path, 'w') as output_file:
      output_file.write(fasta)
      print(f"Finished writing to {output_file_path}")
```

Supplementary data

Table S1 Accession numbers of the sequence data of the included isolates

			Illumina sequencing data			Nanopore sequencing data		
**Name isolate manuscript**	**Name mcr containing plasmid**	**Species**	**Name Sample**	**BioProject no.**	**BioSample no.**	**Name Sample**	**BioProjetct no.**	**BioSample no.**
**A1401**	pMCRESBL1	*Enterobacter cloacae*	PPS066	PRJEB78530	SAMEA115892038	A1401	PRJEB85738	SAMEA117687993
**A1402**	pMCRESBL2	*Citrobacter freundii*	PPS070	PRJEB78530	SAMEA115892042	A1402	PRJEB85738	SAMEA117687994
**A1501**	pMCRESBL3	*Enterobacter cloacae*	PPS105	PRJEB78530	SAMEA115892077	A1501	PRJEB85738	SAMEA117687995
**A1701**	pMCRESBL4	*Enterobacter cloacae*	PPS134	PRJEB78530	SAMEA115892106	A1701	PRJEB85738	SAMEA117687996
**A1702**	pMCRESBL5	*Escherichia coli*	PPS135	PRJEB78530	SAMEA115892107	A1702	PRJEB85738	SAMEA117687997
**A1901**	pMCRESBL6	*Enterobacter cloacae*	PPS150	PRJEB78530	SAMEA115892122	A1901	PRJEB85738	SAMEA117687998
**A2101**	pMCRESBL7	*Enterobacter cloacae*	PPS173	PRJEB78530	SAMEA115892145	A2101	PRJEB85738	SAMEA117687999
**A2201**	pMCRESBL8	*Enterobacter cloacae*	PPS189	PRJEB78530	SAMEA115892161	A2201	PRJEB85738	SAMEA117688000

Table S2 Description of all hits of the database search for *mcr-9* gene containing plasmids

**NUCCORE_Accession**	**NUCCORE_CreateDate**	**NUCCORE_Length**	**NUCCORE_GC**	**TAXONOMY_species**	**TAXONOMY_genus**	**Extended spectrum beta-lactamase genes**	
						Class(es)	Gene(s)
**CP104922.1**	05/06/2023	297,801	0.46	*Citrobacter _portucalensis* (1639133)	*Citrobacter* (544)	ESBL	*bla*$_{CTX-M-3}$
**CP109752.1**	06/06/2023	347,681	0.48	*Enterobacter_hormaechei* (158836)	*Enterobacter* (547)	None	None
**NZ_CP042579.1**	13/10/2019	275,807	0.47	*Enterobacter_kobei* (208224)	*Enterobacter* (547)	ESBL	*bla*$_{SHV-12}$
**NZ_CP083863.1**	13/05/2022	276,053	0.47	*Enterobacter_kobei* (208224)	*Enterobacter* (547)	Carbapenemase, ESBL	*bla*$_{SIM-1}$, *bla*$_{SHV-12}$
**CP109737.1**	06/06/2023	354,602	0.48	*Enterobacter_hormaechei* (158836)	*Enterobacter* (547)	None	None
**NZ_CP109698.1**	07/06/2023	303,280	0.47	*Enterobacter_asburiae* (61645)	*Enterobacter* (547)	ESBL	*bla*$_{SHV-12}$
**CP123704.1**	21/04/2023	257,103	0.45	*Salmonella_enterica* (28901)	*Salmonella* (590)	None	None

**CP123681.1**	20/04/2023	247,657	0.45	*Salmonella_enterica* (28901)	*Salmonella* (590)	None	None
**CP123649.1**	20/04/2023	301,137	0.47	*Salmonella_enterica* (28901)	*Salmonella* (590)	None	None
**NZ_MW244439.1**	07/06/2021	192,620	0.44	*Salmonella_enterica* (28901)	*Salmonella* (590)	None	None
**NZ_MW509820.1**	07/06/2021	249,437	0.48	*Escherichia_coli* (562)	*Escherichia* (561)	None	None
**CP128720.1**	03/07/2023	325,552	0.48	*Klebsiella_pneumoniae* (573)	*Klebsiella* (570)	ESBL	bla_{SHV-12}
**NZ_KY863418.1**	03/06/2020	314,137	0.48	*Enterobacter_asburiae* (61645)	*Enterobacter* (547)	Carbapenemase, ESBL	$bla_{OXA-436}$, bla_{SHV-12}
**NZ_KY978628.1**	03/06/2020	312,880	0.48	*Cronobacter_sakazakii* (28141)	*Cronobacter* (413496)	AmpC, ESBL	bla_{DHA-1}, bla_{SHV-12}
**NZ_MF344583.1**	03/06/2020	349,834	0.48	*Enterobacter_cloacae* (550)	*Enterobacter* (547)	ESBL	bla_{SHV-12}
**NZ_MH399264.1**	03/06/2020	329,420	0.48	*Enterobacter_cloacae* (550)	*Enterobacter* (547)	AmpC, Carbapene	bla_{DHA-1}, bla_{IMP-26}, bla_{SHV-12}

						mase, ESBL	
**NZ_MH829594.1**	03/06/2020	314,351	0.48	*Enterobacter_cloacae* (550)	*Enterobacter* (547)	Carbapenemase, ESBL	*bla*$_{IMP-4}$, *bla*$_{SHV-12}$
**NZ_CP129956.1**	21/07/2023	210,967	0.52	*Enterobacter_asburiae* (61645)	*Enterobacter* (547)	None	None
**CP097199.1**	15/05/2022	298,559	0.48	*Escherichia_coli* (562)	*Escherichia* (561)	None	None
**OW849517.1**	14/05/2022	279,526	0.51	*Enterobacter_cloacae* (550)	*Enterobacter* (547)	None	None
**OW967365.1**	19/05/2022	211,074	0.52	*Enterobacter_cloacae* (550)	*Enterobacter* (547)	None	None
**OW968359.1**	20/05/2022	266,951	0.52	*Enterobacter_cloacae* (550)	*Enterobacter* (547)	None	None
**OW970410.1**	20/05/2022	361,588	0.48	*Enterobacter_cloacae* (550)	*Enterobacter* (547)	None	None
**OW970507.1**	20/05/2022	361,588	0.48	*Enterobacter_cloacae* (550)	*Enterobacter* (547)	None	None

**NZ_CP089439.1**	08/06/2022	231,384	0.48	*Citrobacter _portucalensis* (1639133)	*Citrobacter* (544)	None	None
**NZ_LC542972.1**	04/08/2020	293,502	0.46	*Escherichia_coli* (562)	*Escherichia* (561)	ESBL	*bla*$_{CTX-M-15}$
**NZ_LC542971.1**	04/08/2020	275,138	0.47	*Escherichia_coli* (562)	*Escherichia* (561)	None	None
**NZ_MT077884.1**	04/08/2020	302,192	0.48	*Escherichia_coli* (562)	*Escherichia* (561)	ESBL	*bla*$_{SHV-12}$
**NZ_MT077886.1**	04/08/2020	289,205	0.47	*Escherichia_coli* (562)	*Escherichia* (561)	ESBL	*bla*$_{SHV-12}$
**NZ_MT232840.1**	04/08/2020	312,577	0.48	*Escherichia_coli* (562)	*Escherichia* (561)	Carbapenemase, ESBL	*bla*$_{IMP-4}$, *bla*$_{SHV-12}$
**CP099404.1**	26/06/2022	262,110	0.46	*Klebsiella_pneumoniae* (573)	*Klebsiella* (570)	None	None
**CP101561.1**	25/07/2022	318,780	0.48	*Klebsiella_pneumoniae* (573)	*Klebsiella* (570)	AmpC, Carbapenemase, ESBL	*bla*$_{DHA-1}$, *bla*$_{IMP-26}$, *bla*$_{NDM-1}$, *bla*$_{SHV-12}$

**NZ_CP031102.1**	26/07/2018	221,681	0.47	*Leclercia*_sp._W17 (2282309)	*Leclercia* (83654)	None	None
**NZ_MN423362.1**	15/03/2021	313,802	0.47	*Leclercia*_sp. (1898428)	*Leclercia* (83654)	AmpC, ESBL	bla_{DHA-1}, bla_{SHV-12}
**NZ_CP072971.1**	20/04/2021	342,015	0.47	*Enterobacter*_sp._BWH_37 (1329836)	*Enterobacter* (547)	None	None
**NZ_CP072962.1**	20/04/2021	423,968	0.51	*Enterobacter*_sp._MGH_14 (1329823)	*Enterobacter* (547)	Carbapenemase, ESBL	bla_{KPC-3}, $bla_{SHV-105}$
**NZ_CP091487.1**	03/02/2022	316,592	0.47	*Enterobacter_cloacae*_complex_sp._ECL411 (2912629)	*Enterobacter* (547)	None	None
**CP030718.1**	08/08/2022	296,277	0.47	*Klebsiella_pneumoniae* (573)	*Klebsiella* (570)	ESBL	bla_{SHV-12}
**CP030734.1**	08/08/2022	298,409	0.47	*Klebsiella_pneumoniae* (573)	*Klebsiella* (570)	ESBL	bla_{SHV-12}
**CP030742.1**	08/08/2022	304,705	0.48	*Klebsiella_pneumoniae* (573)	*Klebsiella* (570)	ESBL	bla_{SHV-12}
**NZ_MH909331.1**	04/08/2020	306,267	0.47	*Leclercia_adecarboxylata* (83655)	*Leclercia* (83654)	None	None
**NZ_MK933279.1**	04/08/2020	285,587	0.47	*Enterobacter_hormaechei* (158836)	*Enterobacter* (547)	ESBL	bla_{SHV-12}

**NZ_MN937241.1**	04/08/2020	278,517	0.48	*Enterobacter_cloacae* (550)	*Enterobacter* (547)	None	None
**CP104369.1**	19/09/2022	291,810	0.47	*Salmonella_enterica* (28901)	*Salmonella* (590)	ESBL	*bla*$_{SHV-12}$
**AP026903.1**	15/10/2022	291,417	0.48	*Escherichia_coli* (562)	*Escherichia* (561)	ESBL	*bla*$_{SHV-12}$
**NZ_CP107261.1**	25/10/2022	46,896	0.51	*Leclercia_adecarboxylata* (83655)	*Leclercia* (83654)	None	None
**NZ_CP043767.1**	13/10/2019	331,049	0.48	*Enterobacter_hormaechei* (158836)	*Enterobacter* (547)	None	None
**CP109680.1**	30/10/2022	155,247	0.52	*Enterobacter_cloacae* (550)	*Enterobacter* (547)	None	None
**NZ_CP008825.1**	04/03/2015	282,439	0.47	*Enterobacter_cloacae* (550)	*Enterobacter* (547)	None	None
**NZ_OQ111277.1**	24/07/2023	261,290	0.46	*Enterobacter_hormaechei* (158836)	*Enterobacter* (547)	None	None
**NZ_MF344582.1**	03/06/2020	354,045	0.46	*Citrobacter_freundii* (546)	*Citrobacter* (544)	None	None

**NZ_MN423361.1**	04/08/2020	280,586	0.47	*Leclercia*_sp. (1898428)	*Leclercia* (83654)	ESBL	*bla*$_{SHV-12}$
**NZ_CP091493.1**	03/02/2022	362,923	0.48	*Enterobacter_cloacae*_complex_sp._ECL405 (2912630)	*Enterobacter* (547)	Carbapenemase, ESBL	*bla*$_{IMP-26}$, *bla*$_{SHV-12}$
**NZ_CP091497.1**	03/02/2022	319,000	0.48	*Enterobacter_cloacae*_complex_sp._ECL404 (2912631)	*Enterobacter* (547)	AmpC, ESBL	*bla*$_{DHA-1}$, *bla*$_{SHV-12}$
**NC_012555.1**	14/04/2009	318,782	0.48	*Enterobacter_cloacae* (550)	*Enterobacter* (547)	ESBL	*bla*$_{IMP-8}$, *bla*$_{SHV-12}$
**NZ_CP110355.1**	09/11/2022	286,232	0.47	*Enterobacter_hormaechei* (158836)	*Enterobacter* (547)	None	None
**NZ_CP039390.1**	02/06/2022	337,017	0.48	*Enterobacter_hormaechei* (158836)	*Enterobacter* (547)	AmpC, ESBL	*bla*$_{DHA-1}$, *bla*$_{SHV-12}$
**NZ_CP126799.1**	25/08/2023	339,331	0.47	*Enterobacter_hormaechei* (158836)	*Enterobacter* (547)	None	None
**NZ_CP126812.1**	25/08/2023	248,662	0.46	*Enterobacter_hormaechei* (158836)	*Enterobacter* (547)	None	None
**NZ_CP126820.1**	25/08/2023	249,398	0.46	*Enterobacter_hormaechei* (158836)	*Enterobacter* (547)	None	None

**NZ_CP115001.1**	25/08/2023	283,562	0.47	*Enterobacter_hormaechei* (158836)	*Enterobacter* (547)	ESBL	*bla*$_{SHV-12}$
**NZ_CP126828.1**	25/08/2023	248,621	0.46	*Enterobacter_hormaechei* (158836)	*Enterobacter* (547)	None	None
**NZ_CP133343.1**	08/09/2023	292,549	0.48	*Enterobacter_hormaechei* (158836)	*Enterobacter* (547)	AmpC, ESBL	*bla*$_{DHA-1}$, *bla*$_{SHV-12}$
**NZ_CP133337.1**	08/09/2023	334,536	0.48	*Enterobacter_hormaechei* (158836)	*Enterobacter* (547)	ESBL	*bla*$_{SFO-1}$
**NZ_CP096850.1**	09/09/2023	373,329	0.47	*Enterobacter_kobei* (208224)	*Enterobacter* (547)	None	None
**NZ_CP126835.1**	11/09/2023	335,135	0.48	*Enterobacter_hormaechei* (158836)	*Enterobacter* (547)	AmpC, ESBL	*bla*$_{DHA-7}$, *bla*$_{SHV-12}$
**NZ_CP103643.1**	05/09/2022	188,203	0.52	*Enterobacter_hormaechei* (158836)	*Enterobacter* (547)	ESBL	*bla*$_{SHV-12}$
**NZ_CP083738.1**	29/09/2021	212,640	0.48	*Hafnia_paralvei* (546367)	*Hafnia* (568)	None	None
**NC_012556.1**	14/04/2009	324,503	0.48	*Enterobacter_cloacae* (550)	*Enterobacter* (547)	ESBL	*bla*$_{IMP-8}$, *bla*$_{SHV-12}$
**NZ_CP126832.1**	28/09/2023	332,043	0.48	*Enterobacter_hormaechei* (158836)	*Enterobacter* (547)	AmpC, ESBL	*bla*$_{DHA-7}$, *bla*$_{SHV-12}$

**NZ_CP126838.1**	28/09/2023	335,137	0.48	*Enterobacter_hormaechei* (158836)	*Enterobacter* (547)	AmpC, ESBL	*bla*$_{DHA-7}$, *bla*$_{SHV-12}$
**NZ_CP126816.1**	28/09/2023	248,621	0.46	*Enterobacter_hormaechei* (158836)	*Enterobacter* (547)	None	None
**NZ_CP126825.1**	29/09/2023	248,661	0.46	*Enterobacter_hormaechei* (158836)	*Enterobacter* (547)	None	None
**NZ_CP071389.1**	20/01/2022	295,499	0.48	*Salmonella_enterica* (28901)	*Salmonella* (590)	ESBL	*bla*$_{SHV-12}$
**CP102375.1**	12/10/2023	341,709	0.47	*Leclercia_adecarboxylata* (83655)	*Leclercia* (83654)	None	None
**NZ_CP082281.1**	07/09/2021	327,672	0.47	*Enterobacter*_cancerogenus (69218)	*Enterobacter* (547)	None	None
**LC735981.1**	02/11/2022	177,586	0.53	*Enterobacter_roggenkampii* (1812935)	*Enterobacter* (547)	None	None
**NZ_LT994835.1**	30/06/2018	319,209	0.47	*Klebsiella_pneumoniae* (573)	*Klebsiella* (570)	AmpC, ESBL	*bla*$_{DHA-1}$, *bla*$_{SHV-12}$
**NC_010870.1**	16/06/2008	269,674	0.46	*Klebsiella_pneumoniae* (573)	*Klebsiella* (570)	AmpC, ESBL	*bla*$_{CMY-8}$, *bla*CTX-M-62

**NZ_AP024500.1**	14/03/2021	284,167	0.47	*Enterobacter_asburiae* (61645)	*Enterobacter* (547)	None	None
**NZ_CP031568.1**	09/02/2019	132,677	0.50	*Enterobacter_hormaechei* (158836)	*Enterobacter* (547)	ESBL	*bla*$_{SHV-12}$
**NZ_CP053569.1**	20/01/2022	381,054	0.49	*Enterobacter_cloacae* (550)	*Enterobacter* (547)	None	None
**NZ_CP011596.1**	18/06/2015	113,992	0.52	*Klebsiella_oxytoca* (571)	*Klebsiella* (570)	None	None
**NZ_CP011617.1**	18/06/2015	115,319	0.51	*Klebsiella_oxytoca* (571)	*Klebsiella* (570)	None	None
**NZ_MG288680.1**	03/06/2020	293,643	0.46	*Klebsiella_pneumoniae* (573)	*Klebsiella* (570)	None	None
**NZ_MF788071.1**	03/06/2020	268,242	0.46	*Raoultella_ornithinolytica* (54291)	*Raoultella* (160674)	None	None
**CP086375.1**	05/12/2022	306,935	0.48	*Pseudescherichia_vulneris* (566)	*Pseudescherichia* (2055880)	AmpC, Carbapenemase, ESBL	*bla*$_{DHA-1}$, *bla*$_{IMP-26}$, *bla*$_{SHV-12}$
**NZ_CP094843.1**	08/04/2022	210,249	0.46	*Enterobacter_ludwigii* (299767)	*Enterobacter* (547)	None	None

**NZ_CP032893.1**	21/10/2018	156,442	0.52	*Enterobacter_kobei* (208224)	*Enterobacter* (547)	None	None
**CP085727.1**	01/11/2021	265,587	0.47	*Citrobacter_freundii* (546)	*Citrobacter* (544)	None	None
**NZ_KX810825.1**	03/06/2020	339,962	0.47	*Salmonella_enterica* (28901)	*Salmonella* (590)	None	None
**NZ_CP085755.1**	03/11/2021	289,297	0.48	*Enterobacter_hormaechei* (158836)	*Enterobacter* (547)	ESBL	$bla_{SHV\text{-}12}$
**CP123925.1**	10/05/2023	286,565	0.47	*Salmonella*_sp._SA15303 (3030528)	*Salmonella* (590)	None	None
**CP096599.1**	10/05/2023	345,841	0.48	*Enterobacter_hormaechei* (158836)	*Enterobacter* (547)	AmpC, ESBL	$bla_{DHA\text{-}1}$, $bla_{SHV\text{-}12}$
**CP096993.1**	10/05/2023	282,093	0.47	*Enterobacter_hormaechei* (158836)	*Enterobacter* (547)	None	None
**NZ_CP095662.1**	21/04/2022	276,320	0.47	*Escherichia_coli* (562)	*Escherichia* (561)	None	None
**NZ_OP950838.1**	22/05/2023	285,283	0.47	*Enterobacter_kobei* (208224)	*Enterobacter* (547)	ESBL	$bla_{SHV\text{-}12}$

**NZ_OP950837.1**	22/05/2023	330,692	0.47	*Citrobacter_freundii* (546)	*Citrobacter* (544)	None	None
**NZ_CP064006.1**	22/05/2023	263,699	0.46	*Escherichia_coli* (562)	*Escherichia* (561)	None	None
**NZ_CP117751.1**	18/02/2023	310,787	0.49	*Enterobacter_hormaechei* (158836)	*Enterobacter* (547)	ESBL	*bla*$_{SHV-12}$
**NZ_OP378639.1**	20/02/2023	333,303	0.50	*Klebsiella*_michiganensis (1134687)	*Klebsiella* (570)	None	None
**NZ_KY270852.1**	03/06/2020	282,423	0.47	*Enterobacter_cloacae* (550)	*Enterobacter* (547)	ESBL	*bla*$_{SHV-12}$
**NZ_CP083843.1**	27/04/2022	274,805	0.47	*Enterobacter_asburiae* (61645)	*Enterobacter* (547)	None	None
**NZ_CP118568.1**	03/03/2023	315,525	0.48	*Enterobacter_hormaechei* (158836)	*Enterobacter* (547)	None	None
**NZ_CP047741.1**	11/12/2020	344,276	0.48	*Enterobacter_hormaechei* (158836)	*Enterobacter* (547)	None	None
**NZ_CP066554.1**	26/02/2022	266,565	0.46	*Enterobacter_hormaechei* (158836)	*Enterobacter* (547)	None	None
**NZ_CP029037.1**	02/05/2018	319,930	0.47	*Salmonella_enterica* (28901)	*Salmonella* (590)	None	None

**NZ_CP091126.1**	04/03/2022	295,330	0.47	*Serratia_marcescens* (615)	*Serratia* (613)	ESBL	*bla*$_{SHV-12}$
**NZ_CP012170.1**	17/09/2015	263,138	0.47	*Enterobacter_hormaechei* (158836)	*Enterobacter* (547)	ESBL	*bla*$_{SHV-12}$
**NZ_CP022533.1**	03/08/2017	330,060	0.47	*Enterobacter_hormaechei* (158836)	*Enterobacter* (547)	None	None
**NZ_CP027144.1**	12/03/2018	328,871	0.49	*Enterobacter_hormaechei* (158836)	*Enterobacter* (547)	None	None
**NZ_CP030080.1**	13/07/2018	286,652	0.47	*Enterobacter_hormaechei* (158836)	*Enterobacter* (547)	None	None
**NZ_CP029248.1**	16/08/2018	354,256	0.47	*Enterobacter_hormaechei* (158836)	*Enterobacter* (547)	None	None
**NZ_CP031575.1**	22/08/2018	309,444	0.49	*Enterobacter_hormaechei* (158836)	*Enterobacter* (547)	ESBL	*bla*$_{SHV-12}$
**NZ_CP024910.1**	22/09/2018	351,806	0.47	*Enterobacter_hormaechei* (158836)	*Enterobacter* (547)	None	None
**NZ_CP032842.1**	18/10/2018	339,920	0.48	*Enterobacter_hormaechei* (158836)	*Enterobacter* (547)	ESBL	*bla*$_{SHV-12}$
**NZ_CP042489.1**	13/10/2019	340,181	0.48	*Enterobacter_hormaechei* (158836)	*Enterobacter* (547)	ESBL	*bla*$_{SHV-12}$

**NZ_CP042552.1**	13/10/2019	288,659	0.47	*Enterobacter_hormaechei* (158836)	*Enterobacter* (547)	ESBL	*bla*$_{SHV-12}$
**NZ_CP023570.1**	29/01/2020	291,254	0.47	*Enterobacter_hormaechei* (158836)	*Enterobacter* (547)	None	None
**NZ_CP027112.1**	29/01/2020	291,244	0.47	*Enterobacter_hormaechei* (158836)	*Enterobacter* (547)	None	None
**NZ_CP049047.1**	04/03/2020	322,325	0.48	*Enterobacter_hormaechei* (158836)	*Enterobacter* (547)	AmpC, ESBL	*bla*$_{DHA-1}$, *bla*$_{SHV-12}$
**NZ_CP059425.1**	04/08/2020	302,836	0.48	*Enterobacter_hormaechei* (158836)	*Enterobacter* (547)	ESBL	*bla*$_{SHV-12}$
**NZ_CP059413.1**	04/08/2020	302,551	0.48	*Enterobacter_hormaechei* (158836)	*Enterobacter* (547)	ESBL	*bla*$_{SHV-12}$
**NZ_CP061746.1**	28/09/2020	348,891	0.49	*Enterobacter_hormaechei* (158836)	*Enterobacter* (547)	Carbapenemase, ESBL	*bla*$_{IMP-4}$, *bla*$_{SHV-12}$, *bla*$_{SFO-1}$
**NZ_CP049022.1**	11/12/2020	331,872	0.48	*Enterobacter_hormaechei* (158836)	*Enterobacter* (547)	None	None
**NZ_CP048697.1**	11/12/2020	331,873	0.48	*Enterobacter_hormaechei* (158836)	*Enterobacter* (547)	None	None

**NZ_CP047737.1**	11/12/2020	310,486	0.49	*Enterobacter_hormaechei* (158836)	*Enterobacter* (547)	None	None
**NZ_CP050312.1**	31/01/2021	288,096	0.47	*Enterobacter_hormaechei* (158836)	*Enterobacter* (547)	ESBL	bla_{SHV-12}
**NZ_CP050507.1**	31/01/2021	288,061	0.47	*Enterobacter_hormaechei* (158836)	*Enterobacter* (547)	ESBL	bla_{SHV-12}
**NZ_CP071024.1**	06/03/2021	316,078	0.47	*Enterobacter_hormaechei* (158836)	*Enterobacter* (547)	None	None
**NZ_CP071012.1**	06/03/2021	316,077	0.47	*Enterobacter_hormaechei* (158836)	*Enterobacter* (547)	None	None
**NZ_CP071018.1**	06/03/2021	315,229	0.47	*Enterobacter_hormaechei* (158836)	*Enterobacter* (547)	None	None
**NZ_CP071016.1**	06/03/2021	316,079	0.47	*Enterobacter_hormaechei* (158836)	*Enterobacter* (547)	None	None
**NZ_CP071020.1**	06/03/2021	316,079	0.47	*Enterobacter_hormaechei* (158836)	*Enterobacter* (547)	None	None
**NZ_CP071014.1**	06/03/2021	316,077	0.47	*Enterobacter_hormaechei* (158836)	*Enterobacter* (547)	None	None

**NZ_CP071010.1**	06/03/2021	337,179	0.49	*Enterobacter_hormaechei* (158836)	*Enterobacter* (547)	None	None
**NZ_CP071022.1**	06/03/2021	316,079	0.47	*Enterobacter_hormaechei* (158836)	*Enterobacter* (547)	None	None
**NZ_CP066095.1**	10/02/2022	331,870	0.48	*Enterobacter_hormaechei* (158836)	*Enterobacter* (547)	None	None
**NZ_CP097343.1**	10/08/2022	307,415	0.48	*Enterobacter_hormaechei* (158836)	*Enterobacter* (547)	ESBL	bla_{SHV-12}
**NC_021819.1**	22/07/2013	122,863	0.48	*Salmonella_enterica* (28901)	*Salmonella* (590)	None	None
**CP016526.1**	21/07/2016	261,310	0.47	*Salmonella_enterica* (28901)	*Salmonella* (590)	None	None
**CP022696.1**	14/08/2017	328,945	0.48	*Citrobacter_farmeri* (67824)	*Citrobacter* (544)	None	None
**CP026168.1**	29/01/2018	50,622	0.51	*Leclercia*_sp._LSNIH1 (1920114)	*Leclercia* (83654)	None	None
**CP026661.1**	14/03/2018	310,921	0.48	*Salmonella_enterica* (28901)	*Salmonella* (590)	None	None

**CP028197.1**	02/04/2018	444,417	0.49	*Salmonella_enterica* (28901)	*Salmonella* (590)	ESBL	*bla*$_{SHV-12}$
**CP020529.1**	03/04/2018	400,064	0.48	*Enterobacter_cloacae* (550)	*Enterobacter* (547)	ESBL	*bla*$_{SHV-12}$
**CP027678.1**	25/04/2018	284,485	0.46	*Salmonella_enterica* (28901)	*Salmonella* (590)	None	None
**CP030186.1**	17/07/2018	298,919	0.48	*Salmonella_enterica* (28901)	*Salmonella* (590)	ESBL	*bla*$_{CTX-M-15}$
**CP011601.1**	09/06/2015	295,619	0.47	*Phytobacter_ursingii* (1972431)	*Phytobacter* (447792)	None	None
**CP028975.1**	19/12/2018	340,723	0.49	*Cronobacter_sakazakii* (28141)	*Cronobacter* (413496)	AmpC, ESBL	*bla*$_{DHA-1}$, *bla*$_{SFO-1}$
**CP040696.1**	04/06/2019	309,536	0.48	*Citrobacter_freundii* (546)	*Citrobacter* (544)	AmpC, ESBL	*bla*$_{DHA-1}$, *bla*$_{SHV-12}$
**CP043927.1**	22/09/2019	477,340	0.49	*Klebsiella_quasipneumoniae* (1463165)	*Klebsiella* (570)	AmpC, ESBL	*bla*$_{DHA-1}$, *bla*$_{SHV-12}$
**CP044215.1**	29/09/2019	333,880	0.47	*Klebsiella*_aerogenes (548)	*Klebsiella* (570)	ESBL, Carbapenemase	*bla*$_{SHV-12}$, *bla*$_{IMP-70}$

**CP042506.1**	09/10/2019	357,530	0.47	*Leclercia_adecarboxylata* (83655)	*Leclercia* (83654)	None	None
**CP042494.1**	09/10/2019	357,530	0.47	*Leclercia_adecarboxylata* (83655)	*Leclercia* (83654)	None	None
**CP042525.1**	09/10/2019	339,433	0.47	*Citrobacter_freundii* (546)	*Citrobacter* (544)	None	None
**CP049307.1**	26/02/2020	338,024	0.50	*Salmonella_enterica* (28901)	*Salmonella* (590)	None	None
**CP049309.1**	26/02/2020	310,495	0.45	*Salmonella_enterica* (28901)	*Salmonella* (590)	None	None
**CP049311.1**	26/02/2020	339,705	0.50	*Salmonella_enterica* (28901)	*Salmonella* (590)	None	None
**CP059887.1**	10/08/2020	277,539	0.48	*Salmonella_enterica* (28901)	*Salmonella* (590)	None	None
**CP060516.1**	30/08/2020	278,034	0.48	*Salmonella_enterica* (28901)	*Salmonella* (590)	AmpC, ESBL	$bla_{DHA\text{-}1}$, $bla_{SHV\text{-}12}$
**CP048303.1**	30/04/2020	293,078	0.47	*Salmonella_enterica* (28901)	*Salmonella* (590)	None	None

**CP048299.1**	30/04/2020	293,131	0.47	*Salmonella_enterica* (28901)	*Salmonella* (590)	None	None
**LR890216.1**	30/10/2020	279,735	0.47	*Klebsiella_pneumoniae* (573)	*Klebsiella* (570)	ESBL	*bla*$_{SHV-12}$
**CP029717.1**	10/06/2018	315,949	0.48	*Enterobacter_cloacae*_complex_sp. (2027919)	*Enterobacter* (547)	ESBL	*bla*$_{SHV-12}$
**CP065860.1**	10/12/2020	308,749	0.49	*Salmonella_enterica* (28901)	*Salmonella* (590)	None	None
**CP059429.1**	29/07/2020	369,945	0.49	*Citrobacter_freundii* (546)	*Citrobacter* (544)	None	None
**CP072939.1**	18/04/2021	384,269	0.48	*Klebsiella_pneumoniae* (573)	*Klebsiella* (570)	AmpC, ESBL	*bla*$_{DHA-1}$, *bla*$_{SHV-12}$
**AP024583.1**	20/04/2021	291,362	0.47	*Escherichia_coli* (562)	*Escherichia* (561)	None	None
**CP067381.1**	08/04/2021	297,332	0.48	*Klebsiella_grimontii* (2058152)	*Klebsiella* (570)	None	None
**CP082667.1**	01/09/2021	353,746	0.49	*Salmonella_enterica* (28901)	*Salmonella* (590)	None	None
**CP083235.1**	15/09/2021	320,374	0.48	*Enterobacter_cloacae*_complex_sp. (2027919)	*Enterobacter* (547)	AmpC, Carbapene	*bla*$_{DHA-1}$, *bla*$_{IMP-26}$, *bla*$_{SHV-12}$

						mase, ESBL	
**CP089208.1**	14/12/2021	308,491	0.48	*Salmonella_enterica* (28901)	*Salmonella* (590)	None	None
**CP089412.1**	19/12/2021	269,500	0.47	*Klebsiella_oxytoca* (571)	*Klebsiella* (570)	ESBL	*bla*$_{SHV-12}$
**CP090249.1**	10/01/2022	298,579	0.48	*Escherichia_coli* (562)	*Escherichia* (561)	AmpC, ESBL	*bla*$_{DHA-1}$, *bla*$_{SHV-12}$
**CP082465.1**	02/09/2021	293,413	0.47	*Salmonella_enterica* (28901)	*Salmonella* (590)	ESBL	*bla*$_{SHV-12}$
**CP082418.1**	02/09/2021	276,582	0.47	*Salmonella_enterica* (28901)	*Salmonella* (590)	None	None
**CP082630.1**	01/09/2021	315,781	0.49	*Salmonella_enterica* (28901)	*Salmonella* (590)	None	None
**CP082625.1**	01/09/2021	249,408	0.46	*Salmonella_enterica* (28901)	*Salmonella* (590)	None	None
**CP067079.1**	21/01/2022	271,919	0.47	*Salmonella_enterica* (28901)	*Salmonella* (590)	ESBL	*bla*$_{SHV-12}$
**CP048293.1**	30/04/2020	269,306	0.47	*Escherichia_coli* (562)	*Escherichia* (561)	ESBL	*bla*$_{SHV-12}$

**CP066116.1**	07/02/2022	381,883	0.49	*Citrobacter_freundii* (546)	*Citrobacter* (544)	None	None
**CP083755.1**	18/02/2022	316,459	0.48	*Serratia_marcescens* (615)	*Serratia* (613)	Carbapenemase, ESBL	*bla*$_{\text{IMP-26}}$, *bla*$_{\text{SHV-12}}$
**AP025641.1**	16/03/2022	255,859	0.48	*Citrobacter_koseri* (545)	*Citrobacter* (544)	ESBL	*bla*$_{\text{SHV-12}}$
**AP025654.1**	16/03/2022	256,525	0.48	*Citrobacter_koseri* (545)	*Citrobacter* (544)	ESBL	*bla*$_{\text{SHV-12}}$
**CP091551.1**	11/05/2022	301,285	0.47	*Salmonella_enterica* (28901)	*Salmonella* (590)	AmpC, ESBL	*bla*$_{\text{DHA-1}}$, *bla*$_{\text{CTM-X-3}}$
**NZ_CP008899.1**	21/01/2015	255,013	0.47	*Enterobacter_hormaechei* (158836)	*Enterobacter* (547)	ESBL	*bla*$_{\text{SHV-12}}$
**NZ_CP053104.1**	13/12/2020	149,802	0.52	*Enterobacter_hormaechei* (158836)	*Enterobacter* (547)	None	None
**NZ_CP065940.1**	21/12/2020	307,080	0.47	*Enterobacter_hormaechei* (158836)	*Enterobacter* (547)	ESBL	*bla*$_{\text{SHV-12}}$

**NZ_CP068288.1**	27/01/2021	342,447	0.48	*Enterobacter_hormaechei* (158836)	*Enterobacter* (547)	Carbapenemase, ESBL	*bla*$_{NDM-1}$, *bla*$_{SFO-1}$
**NZ_CP114574.1**	28/12/2022	308,217	0.47	*Enterobacter_hormaechei* (158836)	*Enterobacter* (547)	ESBL	*bla*$_{DHA-1}$, *bla*$_{SHV-12}$
**NZ_CP116348.1**	29/01/2023	296,144	0.52	*Enterobacter_ludwigii* (299767)	*Enterobacter* (547)	None	None
**NZ_CP017930.1**	08/11/2016	114,139	0.51	*Klebsiella_oxytoca* (571)	*Klebsiella* (570)	None	None
**NZ_CP013215.1**	20/04/2020	284,628	0.47	*Klebsiella_pneumoniae* (573)	*Klebsiella* (570)	ESBL	*bla*$_{SHV-12}$
**NZ_MZ156802.1**	17/06/2021	342,942	0.49	*Enterobacter_cloacae*_complex_sp. (2027919)	*Enterobacter* (547)	AmpC, ESBL	*bla*$_{DHA-1}$, *bla*$_{SHV-12}$
**NZ_OK649970.1**	23/08/2022	261,511	0.47	*Citrobacter_freundii* (546)	*Citrobacter* (544)	None	None
**CP042616.1**	15/08/2019	269,463	0.47	*Escherichia_coli* (562)	*Escherichia* (561)	None	None
**CP080514.1**	09/08/2021	352,141	0.48	*Salmonella_enterica* (28901)	*Salmonella* (590)	None	None
**NZ_LC532224.1**	04/08/2020	288,696	0.46	*Enterobacter_hormaechei* (158836)	*Enterobacter* (547)	None	None

**NZ_LC532226.1**	04/08/2020	276,927	0.46	*Enterobacter_hormaechei* (158836)	*Enterobacter* (547)	None	None
**CP067063.1**	23/02/2022	384,723	0.48	*Enterobacter_hormaechei* (158836)	*Enterobacter* (547)	None	None
**NZ_MZ156799.1**	17/06/2021	444,489	0.47	*Enterobacter_cloacae*_complex_sp. (2027919)	*Enterobacter* (547)	AmpC, Carbapenemase, ESBL	bla_{DHA-1}, bla_{NDM-1}, bla_{SHV-12}
**NZ_CP049193.1**	03/03/2020	303,394	0.48	*Enterobacter_hormaechei* (158836)	*Enterobacter* (547)	None	None
**NZ_CP049189.1**	03/03/2020	394,232	0.50	*Enterobacter_hormaechei* (158836)	*Enterobacter* (547)	AmpC, ESBL	bla_{DHA-1}, bla_{SHV-12}, bla_{SFO-1}
**MZ382871.1**	08/09/2021	408,436	0.49	*Enterobacter_cloacae* (550)	*Enterobacter* (547)	ESBL	$bla_{CTX-M-2}$
**NZ_CP083858.1**	22/04/2022	248,708	0.45	*Enterobacter_kobei* (208224)	*Enterobacter* (547)	ESBL	$bla_{CTX-M-9}$
**NZ_OP950833.1**	22/05/2023	276,870	0.46	*Enterobacter_hormaechei* (158836)	*Enterobacter* (547)	ESBL	$bla_{CTX-M-9}$

**NZ_CP091482.1**	03/02/2022	294,412	0.47	*Enterobacter_cloacae*_complex_sp._ECL414 (2912628)	*Enterobacter* (547)	ESBL	*bla*$_{SHV-12}$, *bla*$_{CTX-M-9}$
**NZ_CP050163.1**	31/07/2020	289,268	0.47	*Escherichia_coli* (562)	*Escherichia* (561)	Carbapenemase, ESBL	*bla*$_{KPC-2}$, *bla*$_{SHV-12}$, *bla*$_{CTX-M-14}$
**NZ_CP106895.1**	25/06/2023	262,065	0.46	*Enterobacter_hormaechei* (158836)	*Enterobacter* (547)	None	None
**NZ_CP099763.1**	04/07/2022	287,964	0.47	*Salmonella_enterica* (28901)	*Salmonella* (590)	ESBL	*bla*$_{CTX-M-9}$
**NZ_CP099761.1**	04/07/2022	281,187	0.46	*Salmonella_enterica* (28901)	*Salmonella* (590)	ESBL	*bla*$_{CTX-M-9}$
**NZ_AP022134.1**	13/08/2020	272,100	0.46	*Enterobacter_cloacae* (550)	*Enterobacter* (547)	ESBL	*bla*$_{CTX-M-9}$
**NZ_KP975077.1**	03/06/2020	311,662	0.47	*Enterobacter_cloacae* (550)	*Enterobacter* (547)	Carbapenemase, ESBL	*bla*$_{VIM-1}$, *bla*$_{SFO-1}$, *bla*$_{SHV-12}$, *bla*$_{CTX-M-9}$
**OW849134.1**	14/05/2022	293,564	0.47	*Klebsiella_oxytoca* (571)	*Klebsiella* (570)	ESBL	*bla*$_{SHV-12}$, *bla*$_{CTX-M-9}$

**OW849179.1**	14/05/2022	293,216	0.47	*Enterobacter_cloacae* (550)	*Enterobacter* (547)	Carbapenemase, ESBL	*bla*$_{VIM-1}$, *bla*$_{CTX-M-9}$
**OW849189.1**	14/05/2022	248,724	0.45	*Enterobacter_cloacae* (550)	*Enterobacter* (547)	ESBL	*bla*$_{CTX-M-9}$
**OW849209.1**	14/05/2022	287,088	0.47	*Citrobacter_freundii* (546)	*Citrobacter* (544)	ESBL	*bla*$_{SHV-12}$, *bla*$_{CTX-M-9}$
**OW849257.1**	14/05/2022	290,966	0.47	*Citrobacter_freundii* (546)	*Citrobacter* (544)	ESBL	*bla*$_{SHV-12}$, *bla*$_{CTX-M-9}$
**OW849303.1**	14/05/2022	294,830	0.47	*Enterobacter_cloacae* (550)	*Enterobacter* (547)	Carbapenemase, ESBL	*bla*$_{VIM-1}$, *bla*$_{SHV-12}$, *bla*$_{CTX-M-9}$
**OW849321.1**	14/05/2022	448,858	0.49	*Enterobacter_cloacae* (550)	*Enterobacter* (547)	Carbapenemase, ESBL	*bla*$_{VIM-1}$, *bla*$_{CTX-M-9}$
**OW849357.1**	14/05/2022	294,974	0.47	*Enterobacter_cloacae* (550)	*Enterobacter* (547)	Carbapenemase, ESBL	*bla*$_{VIM-1}$, *bla*$_{CTX-M-9}$

**OW849458.1**	14/05/2022	281,514	0.46	*Enterobacter_cloacae* (550)	*Enterobacter* (547)	ESBL	*bla*$_{CTX-M-9}$
**OW849475.1**	14/05/2022	293,216	0.47	*Enterobacter_cloacae* (550)	*Enterobacter* (547)	Carbapenemase, ESBL	*bla*$_{VIM-1}$, *bla*$_{CTX-M-9}$
**OW849504.1**	14/05/2022	439,447	0.49	*Enterobacter_cloacae* (550)	*Enterobacter* (547)	Carbapenemase, ESBL	*bla*$_{VIM-1}$, *bla*$_{CTX-M-9}$
**OW849533.1**	14/05/2022	290,113	0.47	*Enterobacter_cloacae* (550)	*Enterobacter* (547)	Carbapenemase, ESBL	*bla*$_{VIM-1}$, *bla*$_{CTX-M-9}$
**OW967211.1**	19/05/2022	294,830	0.47	*Enterobacter_cloacae* (550)	*Enterobacter* (547)	Carbapenemase, ESBL	*bla*$_{VIM-1}$, *bla*$_{SHV-12}$, *bla*$_{CTX-M-9}$
**OW968140.1**	20/05/2022	293,216	0.47	*Enterobacter_cloacae* (550)	*Enterobacter* (547)	Carbapenemase, ESBL	*bla*$_{VIM-1}$, *bla*$_{CTX-M-9}$

**OW968418.1**	20/05/2022	281,280	0.46	*Enterobacter_cloacae* (550)	*Enterobacter* (547)	Carbapenemase, ESBL	*bla*$_{VIM-1}$, *bla*$_{CTX-M-9}$
**OW995942.1**	01/06/2022	250,090	0.45	*Citrobacter_freundii* (546)	*Citrobacter* (544)	ESBL	*bla*$_{CTX-M-9}$
**OX030696.1**	02/06/2022	270,215	0.46	*Enterobacter_cloacae* (550)	*Enterobacter* (547)	Carbapenemase, ESBL	*bla*$_{VIM-1}$, *bla*$_{CTX-M-9}$
**CP103665.1**	04/09/2022	271,931	0.47	*Klebsiella*_aerogenes (548)	*Klebsiella* (570)	ESBL	*bla*$_{SHV-12}$, *bla*$_{CTX-M-9}$
**NZ_CP091320.1**	04/02/2022	275,976	0.47	*Enterobacter*_sp._JH25 (2923088)	*Enterobacter* (547)	None	None
**NZ_LT991958.1**	22/03/2018	299,117	0.47	*Enterobacter_cloacae*_complex_sp. (2027919)	*Enterobacter* (547)	None	None
**NZ_CP133857.1**	16/09/2023	299,734	0.46	*Enterobacter_hormaechei* (158836)	*Enterobacter* (547)	Carbapenemase, ESBL	*bla*$_{KPC-2}$, *bla*$_{CTX-M-9}$
**CP136048.1**	09/10/2023	226,955	0.46	*Citrobacter_freundii* (546)	*Citrobacter* (544)	None	None

**NZ_AP022520.1**	18/01/2021	266,476	0.47	*Enterobacter_cloacae* (550)	*Enterobacter* (547)	ESBL	*bla*$_{SHV-12}$
**NZ_CP083831.1**	22/04/2022	373,545	0.49	*Enterobacter_asburiae* (61645)	*Enterobacter* (547)	Carbapenemase, ESBL	*bla*$_{IMP-4}$, *bla*$_{SFO-1}$, *bla*$_{CTX-M-9}$
**NZ_KX710093.1**	03/06/2020	315,395	0.47	*Leclercia_adecarboxylata* (83655)	*Leclercia* (83654)	ESBL	*bla*$_{SFO-1}$
**NZ_CP081340.1**	15/06/2022	324,143	0.47	*Klebsiella_pneumoniae* (573)	*Klebsiella* (570)	ESBL	*bla*$_{CTX-M-9}$
**NZ_OX442404.1**	14/03/2023	277,357	0.46	*Salmonella_enterica* (28901)	*Salmonella* (590)	ESBL	*bla*$_{CTX-M-9}$
**NZ_AP023448.1**	28/01/2021	272,108	0.46	*Enterobacter_roggenkampii* (1812935)	*Enterobacter* (547)	ESBL	*bla*$_{CTX-M-9}$
**NZ_CP083854.1**	22/04/2022	297,683	0.47	*Enterobacter_roggenkampii* (1812935)	*Enterobacter* (547)	ESBL	*bla*$_{SHV-12}$, *bla*$_{CTX-M-9}$
**NZ_CP053645.1**	27/05/2022	295,898	0.47	*Enterobacter_hormaechei* (158836)	*Enterobacter* (547)	ESBL	*bla*$_{SHV-12}$, *bla*$_{CTX-M-9}$
**NZ_CP031724.1**	02/09/2018	296,580	0.47	*Enterobacter_hormaechei* (158836)	*Enterobacter* (547)	ESBL	*bla*$_{SHV-12}$, *bla*$_{CTX-M-9}$

**NZ_CP041734.1**	04/08/2019	276,520	0.46	*Enterobacter_hormaechei* (158836)	*Enterobacter* (547)	Carbapenemase, ESBL	*bla*$_{VIM-4}$, *bla*$_{CTX-M-9}$
**NZ_CP051135.1**	22/04/2020	281,030	0.47	*Enterobacter_hormaechei* (158836)	*Enterobacter* (547)	None	None
**NZ_CP053191.1**	13/05/2020	270,915	0.46	*Enterobacter_hormaechei* (158836)	*Enterobacter* (547)	None	None
**NZ_CP059421.1**	04/08/2020	262,616	0.46	*Enterobacter_hormaechei* (158836)	*Enterobacter* (547)	None	None
**NZ_CP059712.1**	12/08/2020	287,277	0.47	*Enterobacter_hormaechei* (158836)	*Enterobacter* (547)	ESBL	*bla*$_{SHV-12}$
**NZ_CP064657.1**	18/11/2020	267,745	0.46	*Enterobacter_hormaechei* (158836)	*Enterobacter* (547)	ESBL	*bla*$_{CTX-M-9}$
**NZ_AP022511.1**	27/01/2021	292,755	0.47	*Enterobacter_hormaechei* (158836)	*Enterobacter* (547)	ESBL	*bla*$_{CTX-M-9}$
**NZ_CP083850.1**	22/04/2022	283,626	0.47	*Enterobacter_hormaechei* (158836)	*Enterobacter* (547)	ESBL	*bla*$_{SHV-12}$, *bla*$_{CTX-M-9}$
**NZ_CP053693.1**	27/06/2022	274,120	0.46	*Enterobacter_hormaechei* (158836)	*Enterobacter* (547)	ESBL	*bla*$_{CTX-M-9}$

**CP038654.1**	15/11/2019	296,117	0.47	*Citrobacter_freundii* (546)	*Citrobacter* (544)	ESBL, Carbapenemase	*bla*$_{SHV-12}$, *bla*$_{CTX-M-9}$, *bla*$_{VIM-1}$
**CP038657.1**	15/11/2019	263,189	0.46	*Citrobacter_freundii* (546)	*Citrobacter* (544)	ESBL, Carbapenemase	*bla*$_{SHV-12}$, *bla*$_{CTX-M-9}$, *bla*$_{VIM-1}$
**CP038659.1**	15/11/2019	385,971	0.47	*Citrobacter_freundii* (546)	*Citrobacter* (544)	ESBL, Carbapenemase	*bla*$_{SHV-12}$, *bla*$_{CTX-M-9}$, *bla*$_{VIM-1}$
**CP061512.1**	22/09/2020	298,499	0.47	*Mixta_calida* (665913)	*Mixta* (2100764)	ESBL	*bla*$_{SHV-12}$, *bla*$_{CTX-M-9}$
**CP052871.1**	05/05/2020	293,138	0.47	*Enterobacter_cloacae* (550)	*Enterobacter* (547)	ESBL	*bla*$_{SHV-12}$, *bla*$_{CTX-M-9}$
**CP073658.1**	02/05/2021	288,244	0.47	*Klebsiella_quasipneumoniae* (1463165)	*Klebsiella* (570)	None	None
**CP088423.1**	09/12/2021	285,357	0.47	*Escherichia_coli* (562)	*Escherichia* (561)	ESBL	*bla*$_{CTX-M-9}$
**CP089210.1**	14/12/2021	266,098	0.46	*Salmonella_enterica* (28901)	*Salmonella* (590)	None	None

**CP089393.1**	19/12/2021	323,400	0.48	*Klebsiella_oxytoca* (571)	*Klebsiella* (570)	Carbapenemase, ESBL	*bla*$_{IMP-4}$, *bla*$_{SHV-12}$, *bla*$_{CTX-M-9}$
**CP089400.1**	19/12/2021	316,115	0.48	*Klebsiella_oxytoca* (571)	*Klebsiella* (570)	Carbapenemase, ESBL	*bla*$_{IMP-4}$, *bla*$_{CTX-M-9}$
**NZ_MW148604.1**	05/10/2021	237,396	0.46	*Klebsiella_michiganensis* (1134687)	*Klebsiella* (570)	Carbapenemase, ESBL	*bla*SIM-1, *bla*$_{CTX-M-9}$
**NZ_CP110879.1**	23/11/2022	282,472	0.46	*Enterobacter_kobei* (208224)	*Enterobacter* (547)	ESBL	*bla*$_{CTX-M-9}$
**CP136723.1**	23/10/2023	313,339	0.47	*Citrobacter_braakii* (57706)	*Citrobacter* (544)	None	None
**NZ_CP109730.1**	07/06/2023	256,678	0.47	*Enterobacter_roggenkampii* (1812935)	*Enterobacter* (547)	None	None

Table S3 Description of all plasmids (from database and own) belonging to the IncHI group containing *mcr9*, $_{SHV\text{-}12}$ and/or $_{CTX\text{-}M\text{-}9}$

ID	NUCCORE Accession	Sampling date (year)	Sampling location	Bacterial species	ESBL_gene(s)	Replicon(s)	Relaxase(s)	Plasmid **Length** (bp)
**1**	AP025641.1	2012	Japan	*Citrobacter koseri*	*bla*$_{SHV\text{-}12}$	IncHI2A_1, IncHI2_1	unknown	255,859
**2**	AP025654.1	2021	Japan	*Citrobacter koseri*	*bla*$_{SHV\text{-}12}$	IncHI2A_1, IncHI2_1	unknown	256,525
**3**	AP026903.1	2019	Japan:Osaka	*Escherichia coli*	*bla*$_{SHV\text{-}12}$	IncHI2A_1, IncHI2_1	MOBH	291,417
**4**	CP020529.1	2015	USA:Boston	*Enterobacter cloacae*	*bla*$_{SHV\text{-}12}$	IncHI2A_1, IncHI2_1	MOBH	400,064
**5**	CP028197.1	unknown	Czech Republic	*Salmonella enterica*	*bla*$_{SHV\text{-}12}$	IncHI2A_1, IncHI2_1, IncC_1	MOBH,MOBH,MOBH	444,417
**6**	CP029717.1	unknown	unknown	*Enterobacter cloacae complex sp.*	*bla*$_{SHV\text{-}12}$	IncHI2A_1, IncHI2_1	MOBH	315,949
**7**	CP030718.1	2014	Sweden: Gothenburg	*Klebsiella pneumoniae*	*bla*$_{SHV\text{-}12}$	IncHI2A_1, IncHI2_1	MOBH	296,277
**8**	CP030734.1	2013	Sweden: Gothenburg	*Klebsiella pneumoniae*	*bla*$_{SHV\text{-}12}$	IncHI2A_1, IncHI2_1	MOBH	298,409
**9**	CP030742.1	2013	Sweden: Gothenburg	*Klebsiella pneumoniae*	*bla*$_{SHV\text{-}12}$	IncHI2A_1, IncHI2_1	MOBH	304,705
**10**	CP038654.1	2014	Spain	*Citrobacter freundii*	*bla*$_{SHV\text{-}12}$, *bla*$_{CTX\text{-}M\text{-}9}$	IncHI2A_1, IncHI2_1	MOBH	296,117

**11**	CP038657.1	2014	Spain	*Citrobacter freundii*	bla_{SHV-12}, $bla_{CTX-M-9}$	IncHI2A_1, IncHI2_1	MOBH	263,189
**12**	CP038659.1	2014	Spain	*Citrobacter freundii*	bla_{SHV-12}, $bla_{CTX-M-9}$	IncHI2A_1, IncHI2_1	MOBH	385,971
**13**	CP040696.1	2017	China:Wenzhou	*Citrobacter freundii*	bla_{SHV-12}	IncHI2A_1, IncHI2_1	MOBH	309,536
**14**	CP043927.1	2014	Argentina	*Klebsiella quasipneumoniae*	bla_{SHV-12}	IncHI2A_1, IncHI2_1	MOBH,MOBP	477,340
**15**	CP044215.1	2019	United Kingdom:London	*Klebsiella aerogenes*	bla_{SHV-12}	IncHI2A_1, IncHI2_1	MOBH	333,880
**16**	CP048293.1	2018	USA:CA	*Escherichia coli*	bla_{SHV-12}	IncHI2A_1, IncHI2_1	MOBH	269,306
**17**	CP052871.1	2017	Italy: Milan	*Enterobacter cloacae*	bla_{SHV-12}, $bla_{CTX-M-9}$	IncHI2A_1, IncHI2_1	MOBH	293,138
**18**	CP060516.1	2019	Russia	*Salmonella enterica*	bla_{SHV-12}	IncHI2A_1, IncHI2_1	MOBH	278,034
**19**	CP061512.1	2020	Italy: Pavia	*Mixta calida*	bla_{SHV-12}, $bla_{CTX-M-9}$	IncHI2A_1, IncHI2_1	MOBH	298,499
**20**	CP067079.1	2019	China: Guangdong	*Salmonella enterica*	bla_{SHV-12}	IncHI2A_1, IncHI2_1	MOBH	271,919
**21**	CP072939.1	2016	China:Zhejiang	*Klebsiella pneumoniae*	bla_{SHV-12}	IncHI2A_1, IncHI2_1	MOBH	384,269
**22**	CP082465.1	2016	USA:CA	*Salmonella enterica*	bla_{SHV-12}	IncHI2A_1, IncHI2_1	MOBH	293,413

**23**	CP083235.1	2017	China: Jiamusi, Heilongjiang Province	*Enterobacter cloacae* complex sp.	*bla*$_{SHV-12}$	IncHI2A_1, IncHI2_1	MOBH	320,374
**24**	CP083755.1	2021	China:Zhujiang Hospital	*Serratia marcescens*	*bla*$_{SHV-12}$	IncHI2A_1, IncHI2_1	MOBH	316,459
**25**	CP086375.1	2020	China: Zhengzhou	*Pseudescherichia vulneris*	*bla*$_{SHV-12}$	IncHI2A_1, IncHI2_1	MOBH	306,935
**26**	CP088423.1	2016	South Korea	*Escherichia coli*	*bla*$_{CTX-M-9}$	IncHI2A_1, IncHI2_1	MOBH	285,357
**27**	CP089393.1	2018	Australia: Melbourne	*Klebsiella oxytoca*	*bla*$_{SHV-12}$, *bla*$_{CTX-M-9}$	IncHI2A_1, IncHI2_1	MOBH	323,400
**28**	CP089400.1	2018	Australia: Melbourne	*Klebsiella oxytoca*	*bla*$_{CTX-M-9}$	IncHI2A_1, IncHI2_1	MOBH	316,115
**29**	CP089412.1	2018	Australia: Melbourne	*Klebsiella oxytoca*	*bla*$_{SHV-12}$	IncHI2A_1, IncHI2_1	MOBH	269,500
**30**	CP090249.1	2020	China	*Escherichia coli*	*bla*$_{DHA-1}$, *bla*$_{SHV-12}$	IncHI2A_1, IncHI2_1	MOBH	298,579
**31**	CP096599.1	unknown	Hong Kong	*Enterobacter hormaechei*	*bla*$_{DHA-1}$, *bla*$_{SHV-12}$	IncHI2A_1, IncHI2_1	MOBH	345,841

**32**	CP101561.1	2011	China:Kunming	*Klebsiella pneumoniae*	*bla*$_{SHV-12}$	IncHI2A_1, IncHI2_1	MOBH	318,780
**33**	CP103665.1	2018	USA: Houston	*Klebsiella aerogenes*	*bla*$_{SHV-12}$, *bla*$_{CTX-M-9}$	IncHI2A_1, IncHI2_1	MOBH	271,931
**34**	CP104369.1	unknown	USA	*Salmonella enterica*	*bla*$_{SHV-12}$	IncHI2A_1, IncHI2_1	MOBH	291,810
**35**	CP128720.1	unknown	USA: Chicago	*Klebsiella pneumoniae*	*bla*$_{SHV-12}$	IncHI2A_1, IncHI2_1	MOBH	325,552
**36**	LR890216.1	2013	Australia	*Klebsiella pneumoniae*	*bla*$_{SHV-12}$	IncHI2A_1, IncHI2_1	MOBH	279,735
**37**	NC_012555.1	unknown	Taiwan	*Enterobacter cloacae*	*bla*$_{SHV-12}$	IncHI2A_1, IncHI2_1	MOBH	318,782
**38**	NC_012556.1	unknown	Taiwan	*Enterobacter cloacae*	*bla*$_{SHV-12}$	IncHI2A_1, IncHI2_1	MOBH	324,503
**39**	NZ_AP022134.1	2018	Japan:Tokyo	*Enterobacter cloacae*	*bla*$_{CTX-M-9}$	IncHI2A_1, IncHI2_1	MOBH	272,100
**40**	NZ_AP022511.1	2018	Japan:Tokyo	*Enterobacter hormaechei*	*bla*$_{CTX-M-9}$	IncHI2A_1, IncHI2_1	MOBH	292,755
**41**	NZ_AP022520.1	2018	Japan:Tokyo	*Enterobacter cloacae*	*bla*$_{SHV-12}$	IncHI2A_1, IncHI2_1	MOBH	266,476
**42**	NZ_AP023448.1	2019	Japan:Osaka	*Enterobacter roggenkampii*	*bla*$_{CTX-M-9}$	IncHI2A_1, IncHI2_1	MOBH	272,108

**43**	NZ_CP008899.1	2011	USA	*Enterobacter hormaechei* subsp. *hoffmannii* ECNIH3	*bla*$_{SHV-12}$	IncHI2A_1, IncHI2_1	MOBH	255,013
**44**	NZ_CP012170.1	2009	USA	*Enterobacter hormaechei*	*bla*$_{SHV-12}$	IncHI2A_1, IncHI2_1	MOBH	263,138
**45**	NZ_CP013215.1	2010	China:Beijing	*Klebsiella pneumoniae*	*bla*$_{SHV-12}$	IncHI2A_1, IncHI2_1	MOBH	284,628
**46**	NZ_CP031575.1	2015	Missing	*Enterobacter hormaechei*	*bla*$_{SHV-12}$	IncHI2A_1, IncHI2_1	MOBH,MOBP	309,444
**47**	NZ_CP031724.1	2016	China: Sichuan, Chengdu	*Enterobacter hormaechei*	*bla*$_{SHV-12}$, *bla*$_{CTX-M-9}$	IncHI2A_1, IncHI2_1	MOBH	296,580
**48**	NZ_CP032842.1	2007	Australia: Sydney	*Enterobacter hormaechei*	*bla*$_{SHV-12}$	IncHI2A_1, IncHI2_1	MOBH	339,920
**49**	NZ_CP039390.1	2018	China:Jiamusi	*Enterobacter hormaechei*	*bla*$_{SHV-12}$	IncHI2A_1, IncHI2_1	MOBH	337,017
**50**	NZ_CP041734.1	2018	unknown	*Enterobacter hormaechei*	*bla*$_{CTX-M-9}$	IncHI2A_1, IncHI2_1	MOBH	276,520
**51**	NZ_CP042489.1	2009	Australia: Sydney	*Enterobacter hormaechei*	*bla*$_{SHV-12}$	IncHI2A_1, IncHI2_1	MOBH	340,181

**52**	NZ_CP042552.1	2013	Australia: Sydney	*Enterobacter hormaechei*	*bla*$_{SHV-12}$	IncHI2A_1, IncHI2_1	MOBH	288,659
**53**	NZ_CP042579.1	2009	Australia: Sydney	*Enterobacter kobei*	*bla*$_{SHV-12}$	IncHI2A_1, IncHI2_1	MOBH	275,807
**54**	NZ_CP049047.1	2007	China:Wenzhou	*Enterobacter hormaechei*	*bla*$_{SHV-12}$	IncHI2A_1, IncHI2_1	MOBH,MOBH	322,325
**55**	NZ_CP049189.1	2007	China:wenzhou	*Enterobacter hormaechei*	*bla*$_{SHV-12}$, *bla*$_{SFO-1}$	IncHI2A_1, IncHI2_1	MOBH	394,232
**56**	NZ_CP050163.1	2012	Hong Kong	*Escherichia coli*	*bla*$_{SHV-12}$, *bla*$_{CTX-M-14}$	IncHI2A_1, IncHI2_1	MOBH	289,268
**57**	NZ_CP050312.1	2018	Australia:Victoria	*Enterobacter hormaechei*	*bla*$_{SHV-12}$	IncHI2A_1, IncHI2_1	MOBH	288,096
**58**	NZ_CP050507.1	2018	Australia:Victoria	*Enterobacter hormaechei*	*bla*$_{SHV-12}$	IncHI2A_1, IncHI2_1	MOBH	288,061
**59**	NZ_CP053645.1	2017	China: Guangdong	*Enterobacter hormaechei*	*bla*$_{SHV-12}$, *bla*$_{CTX-M-9}$	IncHI2A_1, IncHI2_1	MOBH	295,898
**60**	NZ_CP053693.1	2013	China: Guangdong	*Enterobacter hormaechei*	*bla*$_{CTX-M-9}$	IncHI2A_1, IncHI2_1	MOBH	274,120
**61**	NZ_CP059413.1	2019	Czech Republic: Tabor	*Enterobacter hormaechei*	*bla*$_{SHV-12}$	IncHI2A_1, IncHI2_1	MOBH	302,551

**62**	NZ_CP059425.1	2019	Czech Republic: Ceske Budejovice	*Enterobacter hormaechei*	*bla*$_{SHV-12}$	IncHI2A_1, IncHI2_1	MOBH	302,836
**63**	NZ_CP059712.1	2019	China: Hangzhou	*Enterobacter hormaechei*	*bla*$_{SHV-12}$	IncHI2A_1, IncHI2_1	MOBH	287,277
**64**	NZ_CP061746.1	unknown	China: Jiangsu, Nanjing	*Enterobacter hormaechei*	*bla*$_{SHV-12}$, *bla*$_{SFO-1}$	IncHI2A_1, IncHI2_1	unknown	348,891
**65**	NZ_CP064657.1	2019	China: Hangzhou	*Enterobacter hormaechei*	*bla*$_{CTX-M-9}$	IncHI2A_1, IncHI2_1	MOBH	267,745
**66**	NZ_CP065940.1	2014	Australia: Melbourne	*Enterobacter hormaechei*	*bla*$_{SHV-12}$	IncHI2A_1, IncHI2_1	MOBH	307,080
**67**	NZ_CP071389.1	2018	Germany	*Salmonella enterica*	*bla*$_{SHV-12}$	IncHI2A_1, IncHI2_1	MOBH	295,499
**68**	NZ_CP081340.1	2019	Netherlands	*Klebsiella pneumoniae*	*bla*$_{CTX-M-9}$	IncHI2A_1, IncHI2_1, IncR_1, IncFIA(HI1)_1	MOBH	324,143
**69**	NZ_CP083831.1	2019	China	*Enterobacter asburiae*	*bla*$_{SFO-1}$, *bla*$_{CTX-M-9}$	IncHI2A_1, IncHI2_1	MOBH	373,545
**70**	NZ_CP083850.1	2014	China	*Enterobacter hormaechei*	*bla*$_{SHV-12}$, *bla*$_{CTX-M-9}$	IncHI2A_1, IncHI2_1	MOBH	283,626
**71**	NZ_CP083854.1	2012	China	*Enterobacter roggenkampii*	*bla*$_{SHV-12}$, *bla*$_{CTX-M-9}$	IncHI2A_1, IncHI2_1	MOBH	297,683

**72**	NZ_CP083858.1	2012	China	*Enterobacter kobei*	*bla*$_{CTX\text{-}M\text{-}9}$	IncHI2A_1, IncHI2_1	MOBH	248,708
**73**	NZ_CP083863.1	2011	China	*Enterobacter kobei*	*bla*SIM-1, *bla*$_{SHV\text{-}12}$	IncHI2A_1, IncHI2_1	MOBH	276,053
**74**	NZ_CP085755.1	2020	Czech Republic: South Bohemian Region	*Enterobacter hormaechei*	*bla*$_{SHV\text{-}12}$	IncHI2A_1, IncHI2_1	MOBH	289,297
**75**	NZ_CP091126.1	2015	Australia: U-Vet Werribee Animal Hospital	*Serratia marcescens*	*bla*$_{SHV\text{-}12}$	IncHI2A_1, IncHI2_1	MOBH	295,330
**76**	NZ_CP091482.1	2021	China: Chongqing	*Enterobacter cloacae* complex sp. ECL414	*bla*$_{SHV\text{-}12}$, *bla*$_{CTX\text{-}M\text{-}9}$	IncHI2A_1, IncHI2_1	MOBH	294,412
**77**	NZ_CP091493.1	2021	China: Chongqing	*Enterobacter cloacae* complex sp. ECL405	*bla*$_{SHV\text{-}12}$	IncHI2A_1, IncHI2_1	MOBH	362,923
**78**	NZ_CP091497.1	2020	China: Chongqing	*Enterobacter cloacae* complex sp. ECL404	*bla*$_{SHV\text{-}12}$	IncHI2A_1, IncHI2_1	MOBH	319,000
**79**	NZ_CP097343.1	2018	Germany	*Enterobacter hormaechei*	*bla*$_{SHV\text{-}12}$	IncHI2A_1, IncHI2_1	MOBH	307,415
**80**	NZ_CP099761.1	1995	Belgium	*Salmonella enterica*	*bla*$_{CTX\text{-}M\text{-}9}$	IncHI2A_1, IncHI2_1	MOBH	281,187
**81**	NZ_CP099763.1	2018	Belgium	*Salmonella enterica*	*bla*$_{CTX\text{-}M\text{-}9}$	IncHI2A_1, IncHI2_1	MOBH	287,964

**82**	NZ_CP109698.1	2017	USA	*Enterobacter asburiae*	*bla*$_{SHV-12}$	IncHI2A_1, IncHI2_1	MOBH	303,280
**83**	NZ_CP110879.1	2019	China:Luzhou	*Enterobacter kobei*	*bla*$_{CTX-M-9}$	IncHI2A_1, IncHI2_1	MOBH	282,472
**84**	NZ_CP114574.1	2015	China:a teaching hospital in Henan	*Enterobacter hormaechei*	*bla*$_{SHV-12}$	IncHI2A_1, IncHI2_1	MOBH	308,217
**85**	NZ_CP115001.1	2022	Thailand: Bangkok	*Enterobacter hormaechei*	*bla*$_{SHV-12}$	IncHI2A_1, IncHI2_1	MOBH	283,562
**86**	NZ_CP117751.1	2022	USA	*Enterobacter hormaechei*	*bla*$_{SHV-12}$	IncHI2A_1, IncHI2_1	MOBH,MOBP	310,787
**87**	NZ_CP126832.1	2020	Switzerland	*Enterobacter hormaechei*	*bla*$_{SHV-12}$	IncHI2A_1, IncHI2_1	MOBH	332,043
**88**	NZ_CP126835.1	2020	Switzerland	*Enterobacter hormaechei*	*bla*$_{SHV-12}$	IncHI2A_1, IncHI2_1	MOBH	335,135
**89**	NZ_CP126838.1	2020	Switzerland	*Enterobacter hormaechei*	*bla*$_{SHV-12}$	IncHI2A_1, IncHI2_1	MOBH	335,137
**90**	NZ_CP133343.1	2022	unknown	*Enterobacter hormaechei*	*bla*$_{SHV-12}$	IncHI2A_1, IncHI2_1	unknown	292,549
**91**	NZ_CP133857.1	2022	United Kingdom: England	*Enterobacter hormaechei*	*bla*$_{CTX-M-9}$	IncHI2A_1, IncHI2_1, pXuzhou21_1	MOBH,MOBP	299,734

**92**	NZ_KP975077.1	2015	USA	*Enterobacter cloacae*	*bla*$_{SFO-1}$, *bla*$_{SHV-12}$, *bla*$_{CTX-M-9}$	IncHI2A_1, IncHI2_1	MOBH	311,662
**93**	NZ_KY270852.1	2012	China: Guangzhou	*Enterobacter cloacae*	*bla*$_{SHV-12}$	IncHI2A_1, IncHI2_1, IncR_1	MOBH	282,423
**94**	NZ_KY863418.1	2014	Denmark	*Enterobacter asburiae*	*bla*$_{SHV-12}$	IncHI2A_1, IncHI2_1	MOBH	314,137
**95**	NZ_KY978628.1	2016	China	*Cronobacter sakazakii*	*bla*$_{SHV-12}$	IncHI2A_1, IncHI2_1	MOBH	312,880
**96**	NZ_LT994835.1	2012	France	*Klebsiella pneumoniae*	*bla*$_{SHV-12}$	IncHI2A_1, IncHI2_1	MOBH	319,209
**97**	NZ_MF344583.1	unknown	China	*Enterobacter cloacae*	*bla*$_{SHV-12}$	IncHI2A_1, IncHI2_1, IncR_1	MOBH	349,834
**98**	NZ_MH399264.1	2013	unknown	*Enterobacter cloacae*	*bla*$_{SHV-12}$	IncHI2A_1, IncHI2_1	MOBH	329,420
**99**	NZ_MH829594.1	unknown	China	*Enterobacter cloacae*	*bla*$_{SHV-12}$	IncHI2A_1, IncHI2_1	MOBH	314,351
**100**	NZ_MK933279.1	2018	unknown	*Enterobacter hormaechei*	*bla*$_{SHV-12}$	IncHI2A_1, IncHI2_1, IncR_1	MOBH	285,587
**101**	NZ_MN423361.1	2015	unknown	*Leclercia* sp.	*bla*$_{SHV-12}$	IncHI2A_1, IncHI2_1	MOBH	280,586
**102**	NZ_MN423362.1	2018	unknown	*Leclercia* sp.	*bla*$_{SHV-12}$	IncHI2A_1, IncHI2_1	MOBH	313,802
**103**	NZ_MT077884.1	unknown	unknown	*Escherichia coli*	*bla*$_{SHV-12}$	IncHI2A_1, IncHI2_1	MOBH	302,192

**104**	NZ_MT077886.1	unknown	unknown	*Escherichia coli*	*bla*$_{SHV-12}$	IncHI2A_1, IncHI2_1	MOBH	289,205
**105**	NZ_MT232840.1	2012	unknown	*Escherichia coli*	*bla*$_{SHV-12}$	IncHI2A_1, IncHI2_1	MOBH	312,577
**106**	NZ_MW148604.1	2013	unknown	*Klebsiella michiganensis*	*bla*$_{CTX-M-9}$	IncHI2A_1, IncHI2_1	MOBH	237,396
**107**	NZ_MZ156799.1	2019	unknown	*Enterobacter cloacae* complex sp.	*bla*$_{SHV-12}$	IncHI2A_1, IncHI2_1, IncN_1	MOBF,MOBH	444,489
**108**	NZ_MZ156802.1	2018	unknown	*Enterobacter cloacae* complex sp.	*bla*$_{SHV-12}$	IncHI2A_1, IncHI2_1	MOBH	342,942
**109**	NZ_OP950833.1	2012	Czech Republic: CITYLAB Praha	*Enterobacter hormaechei*	*bla*$_{CTX-M-9}$	IncHI2A_1, IncHI2_1	MOBH	276,870
**110**	NZ_OP950838.1	2012	Czech Republic: Strakonice	*Enterobacter kobei*	*bla*$_{SHV-12}$	IncHI2A_1, IncHI2_1	MOBH	285,283
**111**	NZ_OX442404.1	unknown	unknown	*Salmonella enterica*	*bla*$_{CTX-M-9}$	IncHI2A_1, IncHI2_1	MOBH	277,357
**112**	OW849134.1	2018	Spain	*Klebsiella oxytoca*	*bla*$_{SHV-12}$, *bla*$_{CTX-M-9}$	IncHI2A_1, IncHI2_1	MOBH	293,564
**113**	OW849179.1	2018	Spain	*Enterobacter cloacae*	*bla*$_{CTX-M-9}$	IncHI2A_1, IncHI2_1	MOBH	293,216
**114**	OW849189.1	2018	Spain	*Enterobacter cloacae*	*bla*$_{CTX-M-9}$	IncHI2A_1, IncHI2_1	MOBH	248,724
**115**	OW849209.1	2018	Spain	*Citrobacter freundii*	*bla*$_{SHV-12}$, *bla*$_{CTX-M-9}$	IncHI2A_1, IncHI2_1	MOBH	287,088

**116**	OW849257.1	2018	Spain	*Citrobacter freundii*	*bla*$_{SHV-12}$, *bla*$_{CTX-M-9}$	IncHI2A_1, IncHI2_1	MOBH	290,966
**117**	OW849303.1	2018	Spain	*Enterobacter cloacae*	*bla*$_{SHV-12}$, *bla*$_{CTX-M-9}$	IncHI2A_1, IncHI2_1	MOBH	294,830
**118**	OW849321.1	2018	Spain	*Enterobacter cloacae*	*bla*$_{CTX-M-9}$	IncHI2A_1, IncHI2_1, IncFII(pECLA)_1, IncFIB(pECLA)_1	MOBF,MOBH	448,858
**119**	OW849357.1	2018	Spain	*Enterobacter cloacae*	*bla*$_{CTX-M-9}$	IncHI2A_1, IncHI2_1	MOBH	294,974
**120**	OW849458.1	2018	Spain	*Enterobacter cloacae*	*bla*$_{CTX-M-9}$	IncHI2A_1, IncHI2_1	MOBH	281,514
**121**	OW849475.1	2018	Spain	*Enterobacter cloacae*	*bla*$_{CTX-M-9}$	IncHI2A_1, IncHI2_1	MOBH	293,216
**122**	OW849504.1	2018	Spain	*Enterobacter cloacae*	*bla*$_{CTX-M-9}$	IncHI2A_1, IncHI2_1, IncFII(pECLA)_1, IncFIB(pECLA)_1	MOBF,MOBH	439,447
**123**	OW849533.1	2018	Spain	*Enterobacter cloacae*	*bla*$_{CTX-M-9}$	IncHI2A_1, IncHI2_1	MOBH	290,113
**124**	OW967211.1	2018	Spain	*Enterobacter cloacae*	*bla*$_{SHV-12}$, *bla*$_{CTX-M-9}$	IncHI2A_1, IncHI2_1	MOBH	294,830
**125**	OW968140.1	2018	Spain	*Enterobacter cloacae*	*bla*$_{CTX-M-9}$	IncHI2A_1, IncHI2_1	MOBH	293,216
**126**	OW968418.1	2018	Spain	*Enterobacter cloacae*	*bla*$_{CTX-M-9}$	IncHI2A_1, IncHI2_1	MOBH	281,280
**127**	OW995942.1	2018	Spain	*Citrobacter freundii*	*bla*$_{CTX-M-9}$	IncHI2A_1, IncHI2_1	MOBH	250,090

**128**	OX030696.1	2018	Spain	*Enterobacter cloacae*	*bla*$_{CTX\text{-}M\text{-}9}$	IncHI2A_1, IncHI2_1	MOBH	270,215
**129**	pMCRESBL1	2014	Netherlands: Breda	*Enterobacter cloacae* complex	*bla*$_{SHV\text{-}12}$, *bla*$_{CTX\text{-}M\text{-}9}$	IncHI2A_1, IncHI2_1	MOBH	268,712
**130**	pMCRESBL2	2014	Netherlands: Breda	*Citrobacter freundii*	*bla*$_{CTX\text{-}M\text{-}9}$	IncHI2A_1, IncHI2_1	MOBH	242,886
**131**	pMCRESBL3	2015	Netherlands: Breda	*Enterobacter cloacae* complex	*bla*$_{SHV\text{-}12}$, *bla*$_{CTX\text{-}M\text{-}9}$	IncHI2A_1, IncHI2_1	MOBH	304,453
**132**	pMCRESBL4	2017	Netherlands: Breda	*Enterobacter cloacae* complex	*bla*$_{SHV\text{-}12}$, *bla*$_{CTX\text{-}M\text{-}9}$	IncHI2A_1, IncHI2_1	MOBH	282,195
**133**	pMCRESBL5	2017	Netherlands: Breda	*Escherichia coli*	*bla*$_{CTX\text{-}M\text{-}9}$	IncHI2A_1, IncHI2_1	MOBH	242,975
**134**	pMCRESBL6	2019	Netherlands: Breda	*Enterobacter cloacae* complex	*bla*$_{SHV\text{-}12}$, *bla*$_{CTX\text{-}M\text{-}9}$	IncHI2A_1, IncHI2_1	MOBH	274,716
**135**	pMCRESBL8	2022	Netherlands: Breda	*Enterobacter cloacae* complex	*bla*$_{SHV\text{-}12}$, *bla*$_{CTX\text{-}M\text{-}9}$	IncHI2A_1, IncHI2_1	MOBH	278,511

Table S4 Inferred clock rates expressed as substitutions per base pair per year in the IncHI plasmid background based on the models with a strict clock.

Model	Mean	Median	95% HPD
**Coalescent constant population**	1.06E-06	1.06E-06	7.56E-07 - 1.39E-06
**Coalescent exponential population**	9.99E-07	9.98E-07	6.93E-07 - 1.31E-06
**Coalescent bayesian skyline**	8.94E-07	8.94E-07	6.44E-07 - 1.13E-06

Supplementary figures

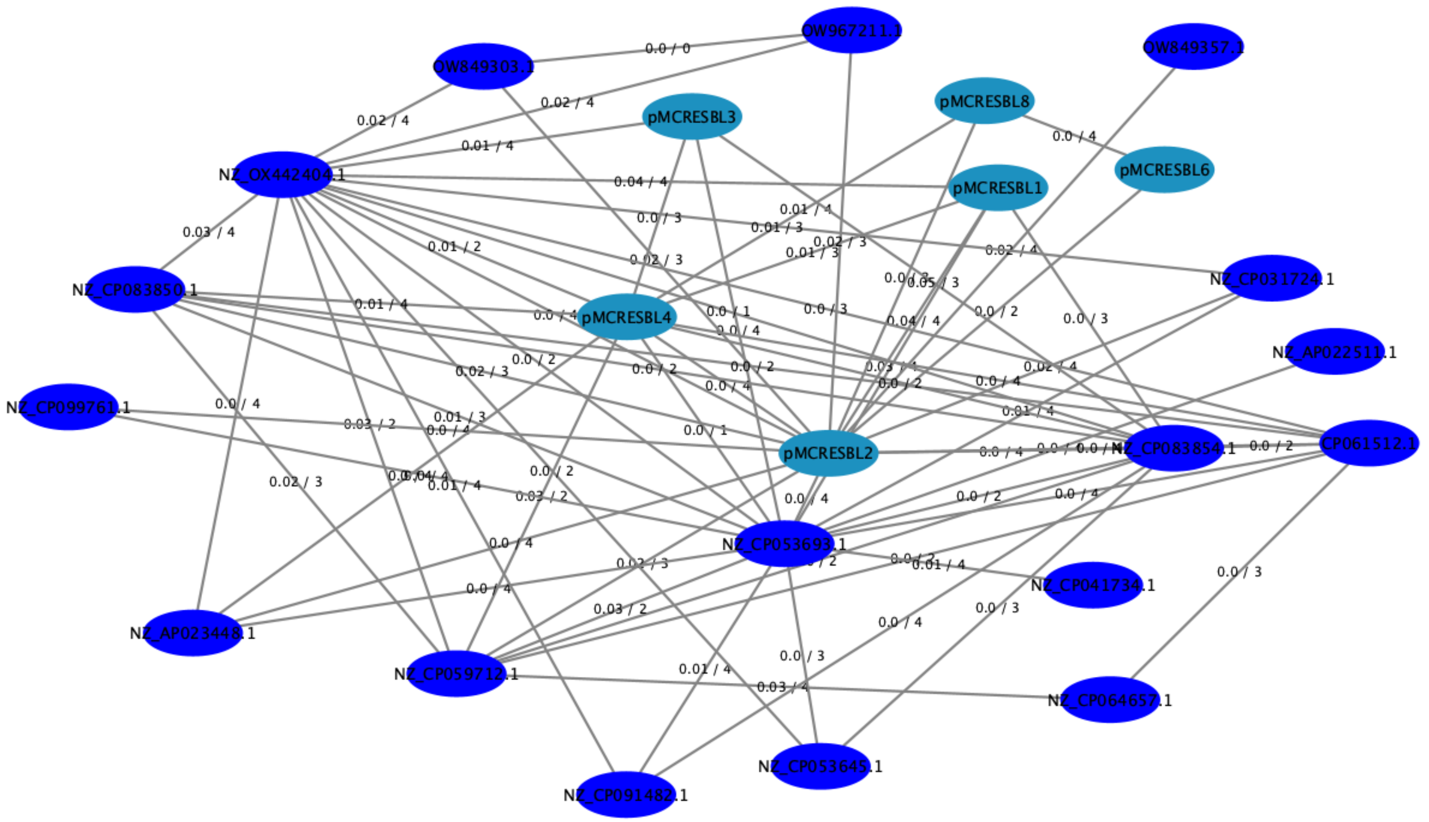

Figure S1 The relatedness network constructed using Pling containing the PPS study plasmids and related database plasmids with a maximum number of four structural events apart between connected plasmids. The blue nodes are plasmids (light blue PPS study plasmids, dark blue database plasmids), and edges are labeled with two numbers: 1) the containment distance (what proportion of the smaller plasmid is not alignable to the larger plasmid) and 2) the rearrangement distance / Double Cut and Join Indel (DCJ-Indel) distance (how many rearrangements/indels separate these two plasmids). pMCRESBL5 is not visualized in this network, because this plasmid was identified as a hub plasmid and thus excluded to prevent overclustering based on transposible elements.

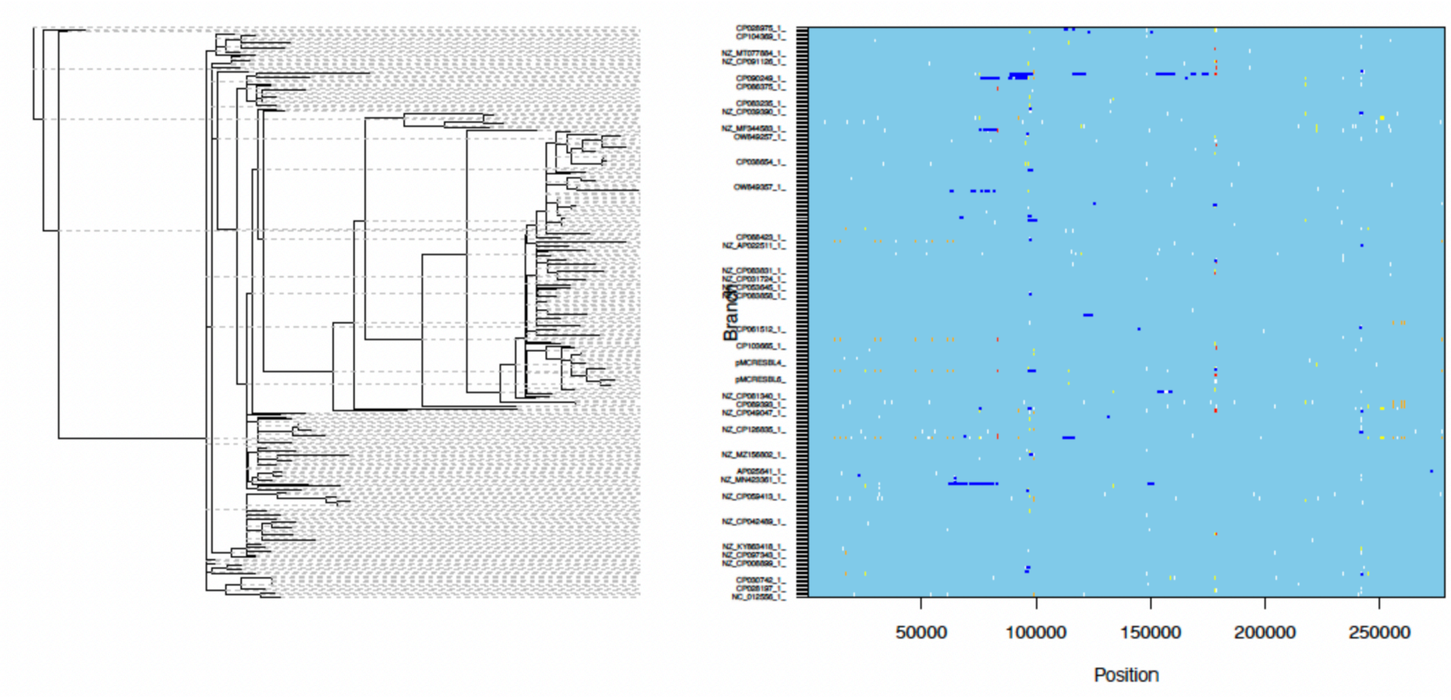

Figure S2 ClonalFrameML output with in dark blue the regions suspected of recombination events

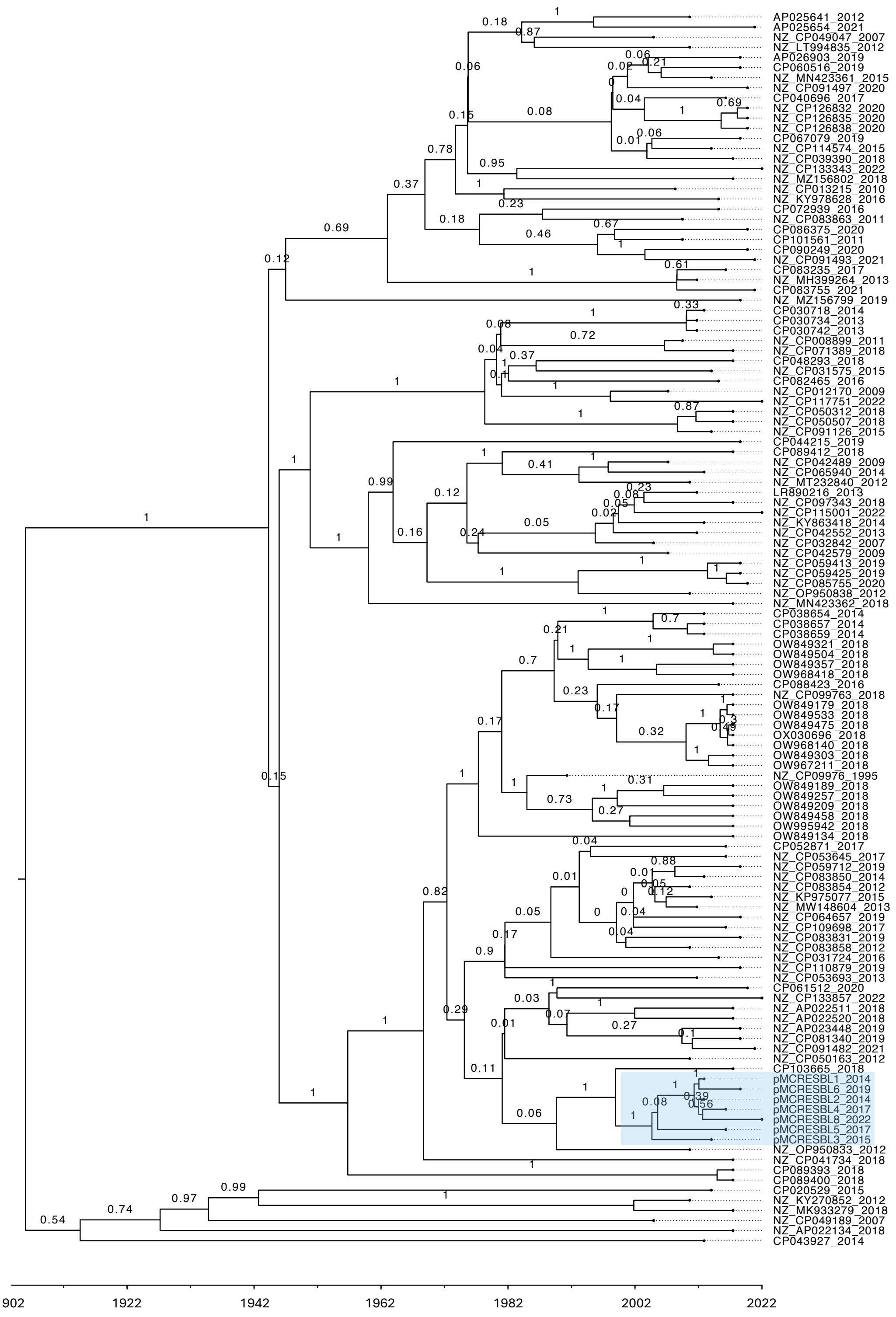

Figure S3 Time-scaled maximum clade credibility (MCC) tree based on inferred time phylogeny according to the coalescent constant population model with a strict clock of all IncHI plasmids containing *mcr-9*, *bla*$_{\text{SHV-12}}$ and/or *bla*$_{\text{CTX-M-9}}$. The PPS study plasmids are highlighted in blue and the branches are annotated with the posterior probability. The time in years at the bottom of the tree represents the year of the origin of the nodes.

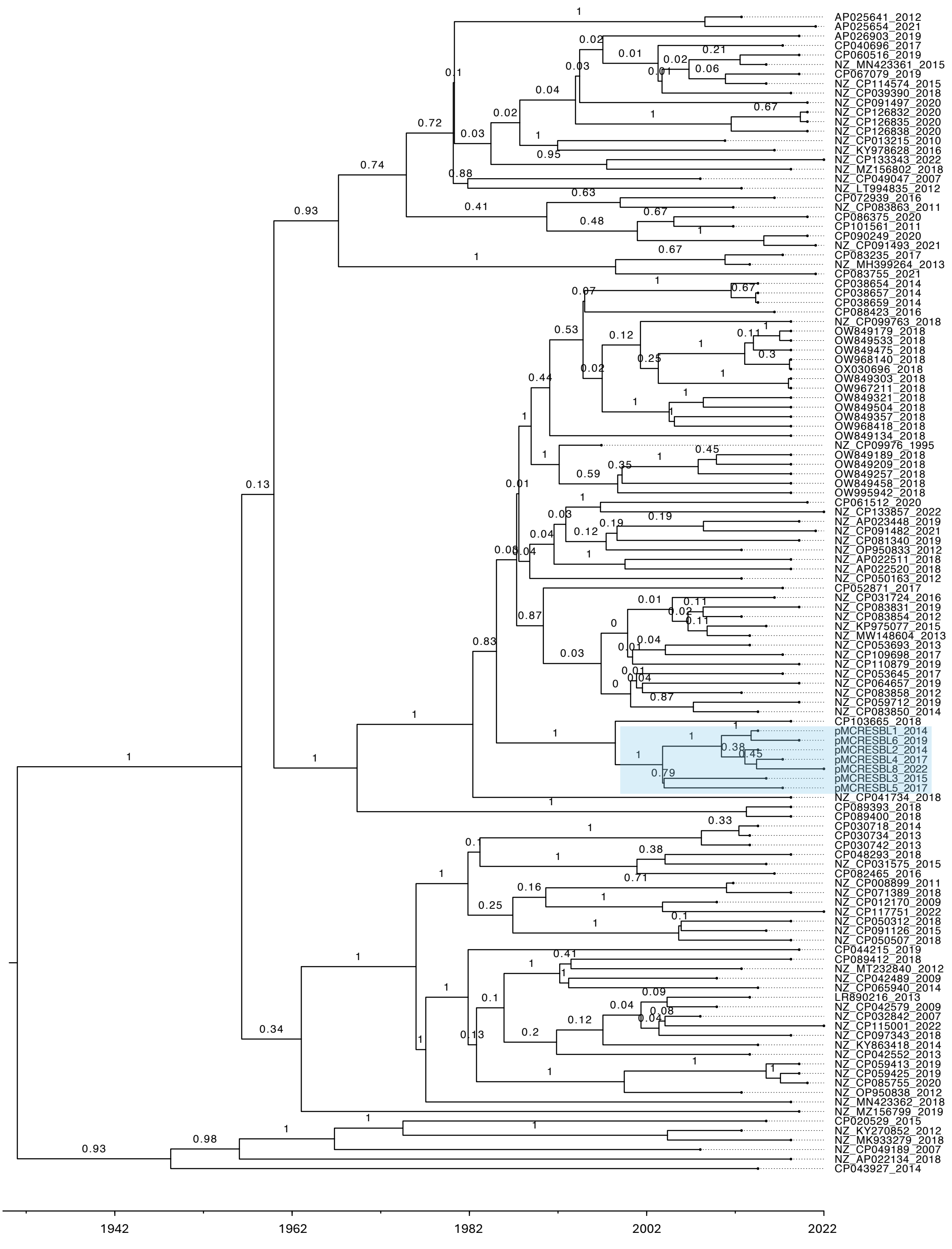

Figure S4 Time-scaled maximum clade credibility (MCC) tree based on inferred time phylogeny according to the coalescent exponential population model with a strict clock of all IncHI plasmids containing *mcr-9*, *bla*$_{SHV-12}$ and/or *bla*$_{CTX-M-9}$. The PPS study plasmids are highlighted in blue and the branches are annotated with the posterior probability. The time in years at the bottom of the tree represents the year of the origin of the nodes.

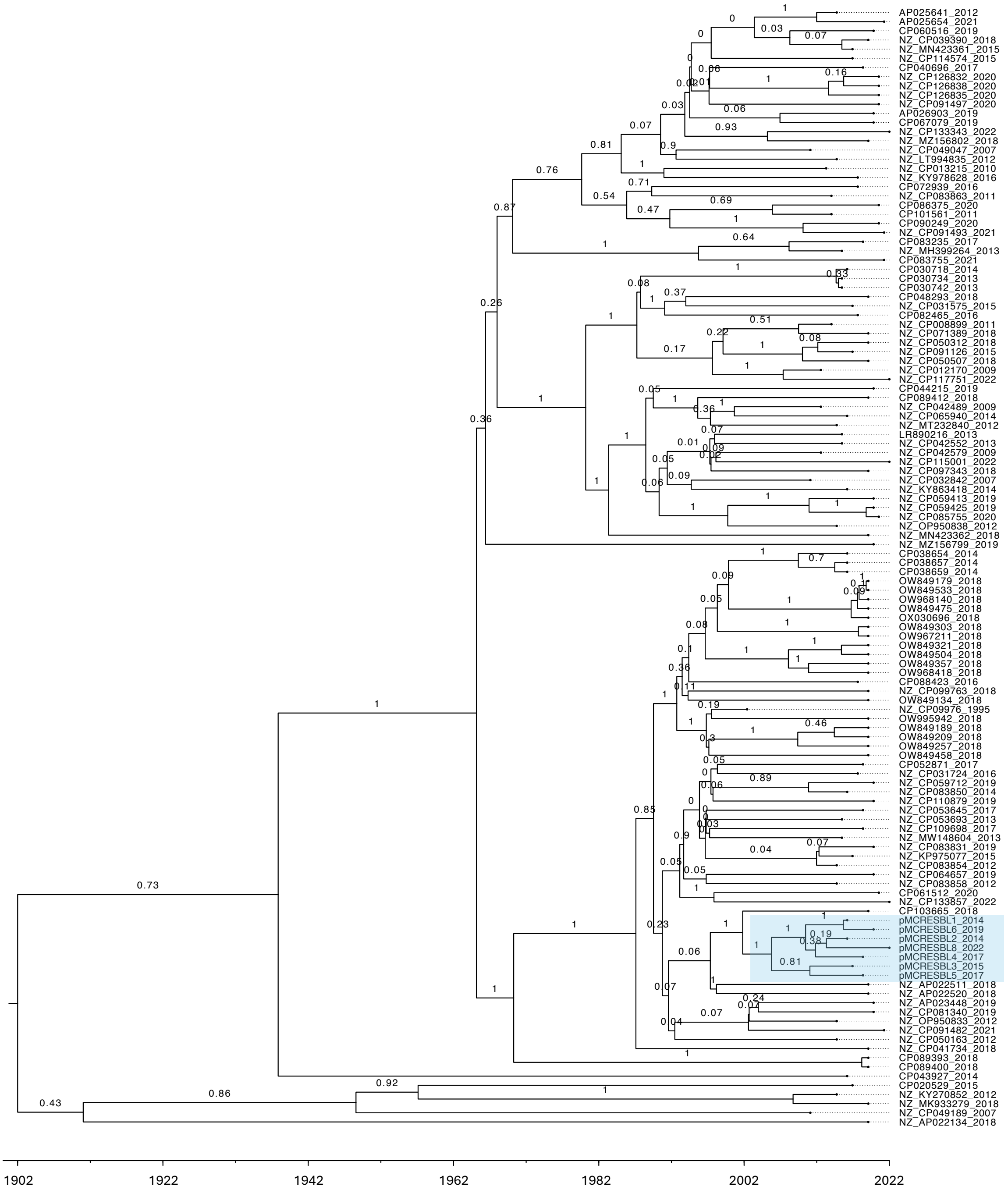

Figure S5 Time-scaled maximum clade credibility (MCC) tree based on inferred time phylogeny according to the coalescent Bayesian skyline model with a strict clock of all IncHI plasmids containing *mcr-9*, *bla*$_{SHV-12}$ and/or *bla*$_{CTX-M-9}$. The PPS study plasmids are highlighted in blue and the branches are annotated with the posterior probability. The time in years at the bottom of the tree represents the year of the origin of the nodes.

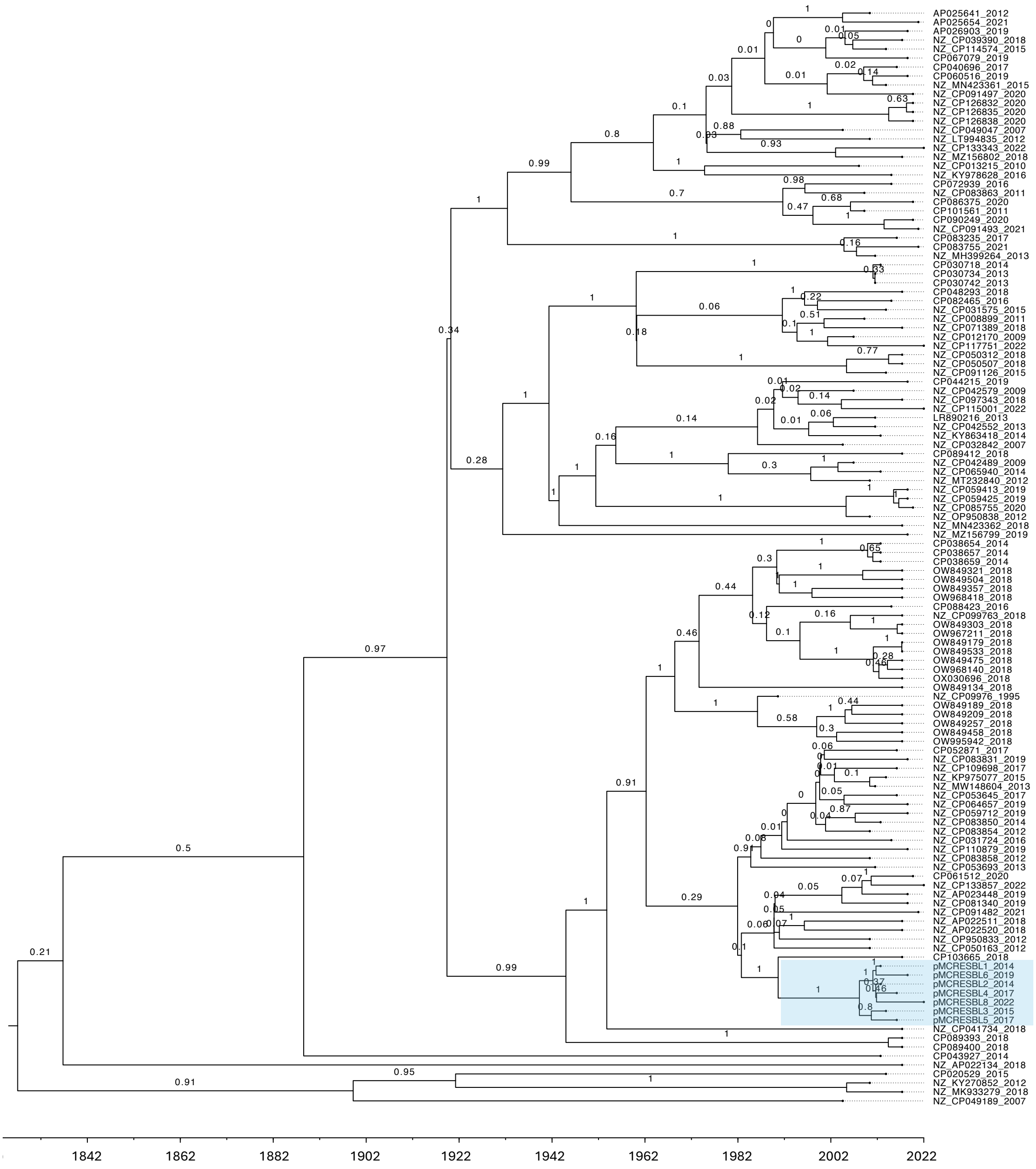

Figure S6 Time-scaled maximum clade credibility (MCC) tree based on inferred time phylogeny according to the coalescent constant population model with an optimised relaxed clock of all IncHI plasmids containing *mcr-9*, *bla*$_{SHV-12}$ and/or *bla*$_{CTX-M-9}$. The PPS study plasmids are highlighted in blue and the branches are annotated with the posterior probability. The time in years at the bottom of the tree represents the year of the origin of the nodes.

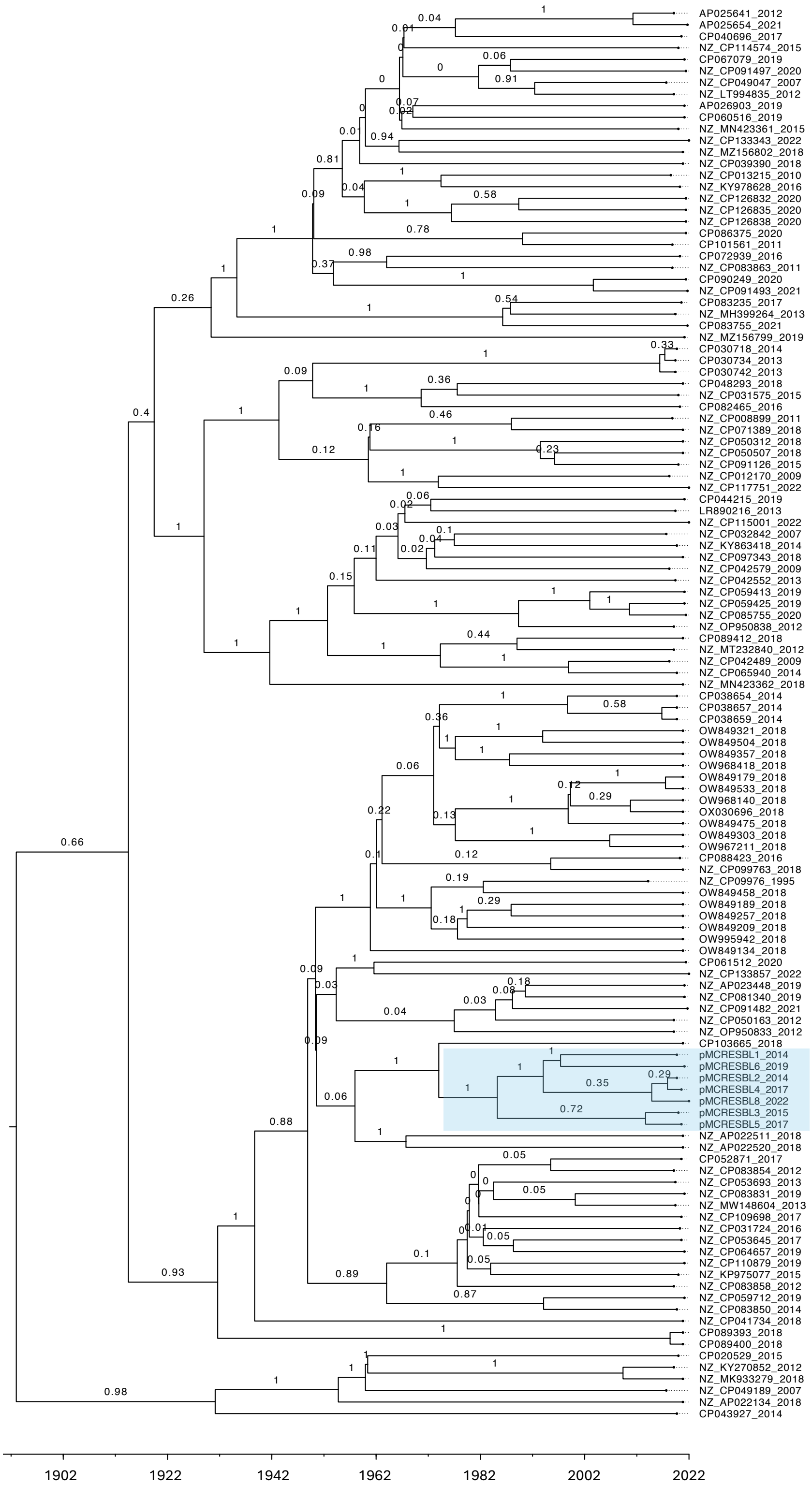

Figure S7 Time-scaled maximum clade credibility (MCC) tree based on inferred time phylogeny according to the coalescent exponential population model with an optimised relaxed clock of all IncHI plasmids containing *mcr-9*, *bla*$_{SHV-12}$ and/or *bla*$_{CTX-M-9}$. The PPS study plasmids are highlighted in blue and the branches are annotated with the posterior probability. The time in years at the bottom of the tree represents the year of the origin of the nodes.

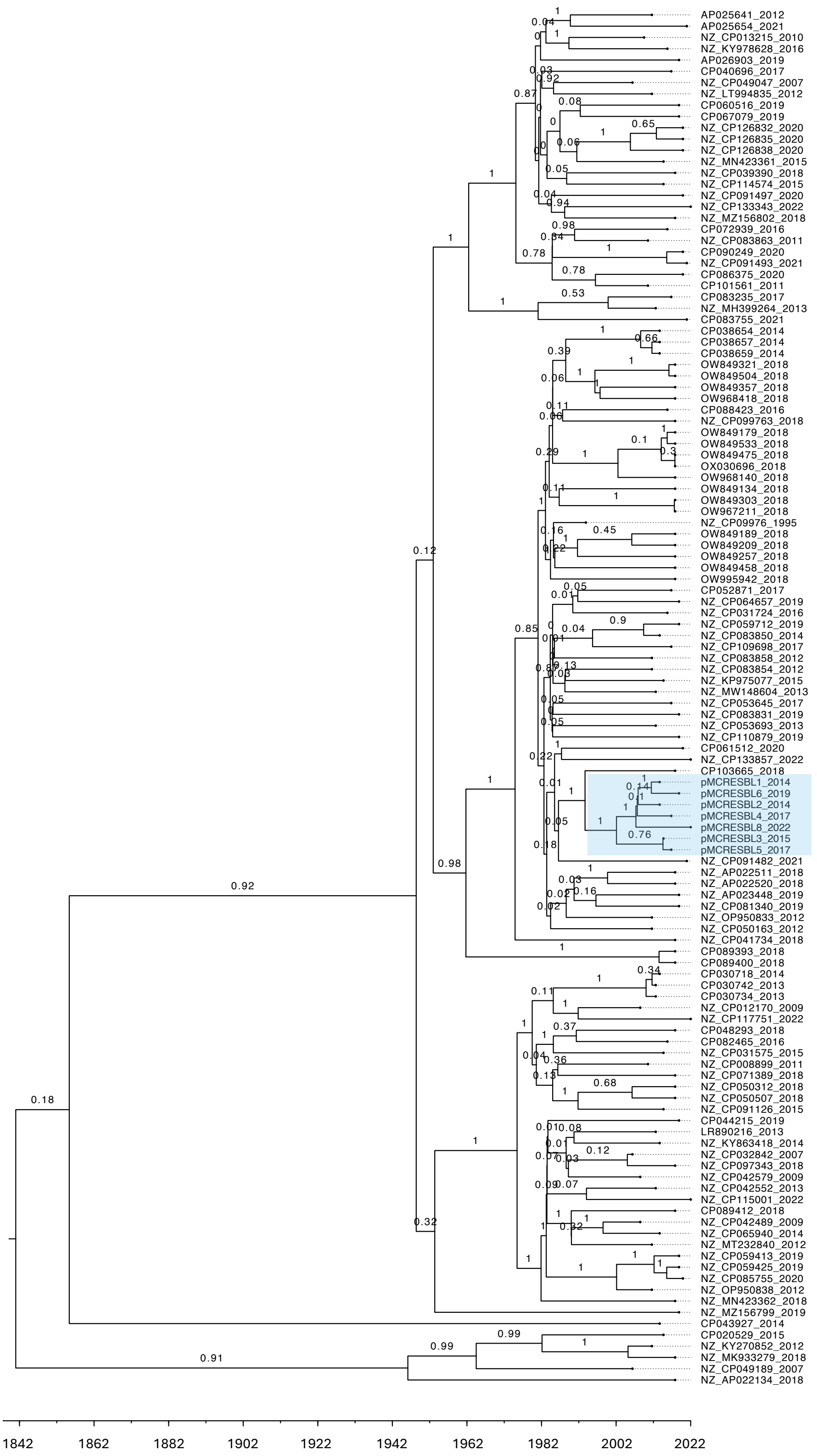

Figure S8 Time-scaled maximum clade credibility (MCC) tree based on inferred time phylogeny according to the coalescent Bayesian skyline model with an optimised relaxed clock of all IncHI plasmids containing *mcr-9*, *bla*$_{SHV-12}$ and/or *bla*$_{CTX-M-9}$. The PPS study plasmids are highlighted in blue and the branches are annotated with the posterior probability. The time in years at the bottom of the tree represents the year of the origin of the nodes.

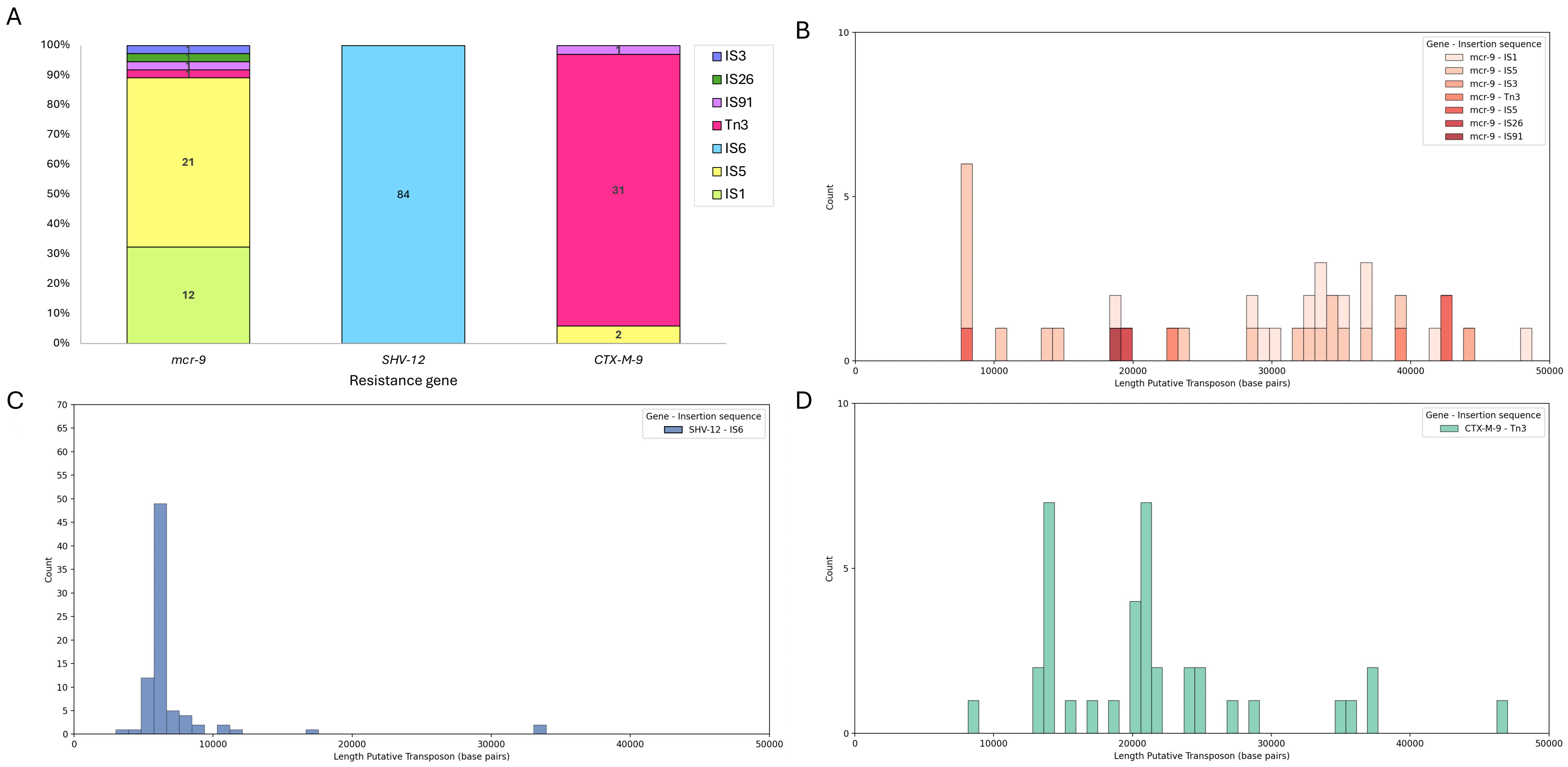

Figure S9 Putative transposases defined as stretches of DNA flanked insertion sequences <50 Kb apart around the genes of interest. In B, C and D the length the putative composite transposons of *mcr-9.1*, *bla*$_{\text{SHV-12}}$ and *bla*$_{\text{CTX-M-9}}$, respectively.
